# Supplementary material for: Learning meaningful representations of protein sequences
Source: Nat Commun. 2022 Apr 8;13:1914. doi: 10.1038/s41467-022-29443-w (PMC8993921; doi:10.1038/s41467-022-29443-w)
Supplement: Supplementary file 1 — Supplementary Information [file 41467_2022_29443_MOESM1_ESM.pdf]

Supplementary Material: Learning meaningful representations of protein sequences

Nicki Skafte Detlefsen
Section for Cognitive Systems
Technical University of Denmark

Søren Hauberg
Section for Cognitive Systems
Technical University of Denmark

Wouter Boomsma
Department of Computer Science
University of Copenhagen
wb@di.ku.dk

Supplementary Methods

Datasets. In the transfer-learning experiments we use 31 million protein sequences extracted from the Pfam database [1], following the procedure described for the TAPE benchmark set [2]. We use data for remote homology detection from [3], fluorescence landscape prediction from [4] and for stability landscape prediction from [5], all spanning multiple protein families. See Table 1 for the specific dataset sizes.

For the experiments regarding the analysis of reconstruction error as a measure of downstream performance (1), we use the UniLanguage dataset [6]. UniLanguage consists of samples from the UniProt [7] database, where splits were constructed to minimize the overlap between families.

For the study of latent space structure on single protein families we consider the β-lactamase sequences extracted from Pfam [1], family PF00144, where we also obtain a sequence alignment. For the study of subclasses of beta-lactamase, we use the beta-lactamase alignment provided in the DeepSequence study [8], which is processed in different ways as described in the main paper. The processing scripts are provided as part of the source code repository associated with our manuscript.

Task	Train	Valid	Test
Language Mod.	32,207,059	N/A	44,314
Unilanguage [6]	607,737	98,907	295,161
Remote Homol.	12,312	736	718
Fluorescence	21,446	5,362	27,217
Stability	53,679	2,447	12,839

Supplementary Table 1: Data set sizes used for the prediction tasks (i.e. the transfer-learning setting).

Predictive tasks. In the transfer-learning experiments, the Transformer and Resnet based models were pre-trained using a masked token prediction task [9] where 15% of the amino acids in a sequence are masked out and the task is to predict the identity of the masked amino acids from the non-masked. The LSTM model were trained using next-token prediction, where the task is to predict the next amino acid in a sequence given the amino acids processed until now. Lastly, the autoencoder (bottleneck) models were trained with standard reconstruction tasks. Details for the three downstream tasks are listed below:

1. Fluorescence: An input protein sequence $\mathbf{s}$ is mapped to a label $y \in \mathbb{R}$ corresponding to the log-fluorescence intensity of $\mathbf{s}$, that expresses a models ability to distinguish between similar sequences. The models are optimized using the mean squared loss and performance is measured using Spearman correlation.

2. Stability: An input protein sequence $\mathbf{s}$ is mapped to a label $y \in \mathbb{R}$ corresponding to the most extreme value for which the protein keeps its fold. The models are optimized using the mean squared loss and performance is measured using Spearman correlation.

3. Remote homology: An input protein sequence $\mathbf{s}$ is mapped to a label $y \in \{1, ..., 1195\}$, where each class correspond to a specific protein fold. The models are optimized using categorical cross entropy and performance is measured using accuracy.

Resnet, LSTM, Transformer

The Resnet, LSTM and Transformer architectures used in the first experiments are all directly taken from TAPE [2]. The Transformer consist of 12-layers with a hidden size of 512 and 8 attention heads, which leads to a 38M-parameter model. The architectures of the two other models were chosen such that the total number of parameters match that of the Transformer. In this case, the Resnet consist of 35 layers each with 256 filters, a dilation rate of 2

and a kernel size of 9. The LSTM has 3 bidirectional layers each with 1024 hidden units. If not stated otherwise, we use an attention based aggregation function for combining the local representations into a single global representation.

Bottleneck AutoEncoder

For the Bottleneck Resnet autoencoder we used an encoder-decoder architecture where both the encoder and decoder were modeled using resnet blocks. In contrast to the three models above, the bottleneck autoencoder is not a sequential model and requires a fixed size input. All sequences were therefore padded with zeros to the same length (3000). For the encoder we use 30 residual blocks with pooling along the sequence dimension every 5 layer. For the decoder we inverse the process and again use 30 residual blocks, this time with upsampling along the sequence dimension every 5 layer. Between the encoder and decoder we had two fully connected layers that respectively downsampled and upsampled from the global latent space. The AutoEncoder has approximately twice the number of parameters as the sequential models during pre-training, but was designed to have the same number of parameters when used for transfer-learning as the decoder is disabled in this setting.

VAE

The architecture of the VAE is a simplified version of that used in the DeepSequence paper [8], using an encoder with two fully connected hidden layers (both with 1500 nodes) with ReLU activations, and a decoder with two fully connected hidden layers (100 and 500 nodes) also with ReLU activations.

Training details. We followed the training protocol from [2] for pretraining on Pfam and training of the task specific models. Pre-training was performed on four NVIDIA TITAN V GPUs for 1 week. Hyperparameters were set as follows:

- Adam optimizer was used with default settings for momentum.
- Learning rate: initialized to 10^{-3}, adjusted using a linear warm-up scheduler.
- 10% dropout rate.
- Batch size was dynamically set during training to the largest possible based on model architecture and sequence length.

Task-specific training was performed using the same set of GPUs and hyperparameters, but training was stopped early when no increase in validation performance was observed. If not stated otherwise, we always complete pre-training before task-specific training to get the best possible performing model.

For the training on β-lactamase model we deploy nearly the same training strategy as with the Pfam family, however with extra steps to prevent overfitting, which is more likely on a single family than the full corpus of proteins. In particular we use early stopping monitored on the validation loss with a patience of 10 epochs to ensure that we do not use highly overfitted models.

The VAEs were trained using the same optimizer settings, but with a fixed learning rate, no dropout and using a fixed batch size of 16.

Supplementary Results

Reconstruction accuracy is not a good proxy for downstream performance

As stated in the main paper, we observe that reconstruction accuracy may be a poor proxy for the quality of the representation itself, as it does not directly correlate with the downstream performance metrics (Supplementary Figure 1). To further investigate the phenomenon, we re-trained a number of embedding models, where we gradually lowered the amount of pre-training data available to the model (Supplementary Figure 2). We again observe a discrepancy between the reconstruction performance and the downstream performance metrics, with the

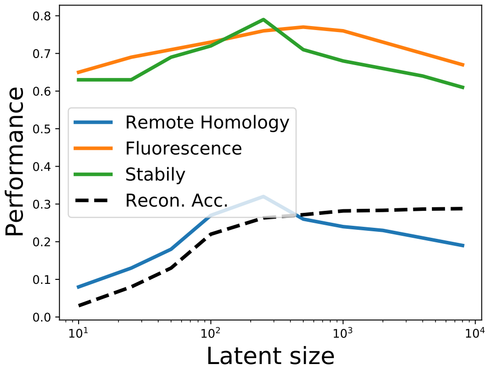

Supplementary Figure 1: Reconstruction and downstream performance as a function of representation size. Although reconstruction accuracy consistently improves for increasing representation size, the performances on the individual tasks deteriorate for large representations. Performance refers to Spearman correlation (stability, fluorescence) or accuracy (homology, reconstruction).

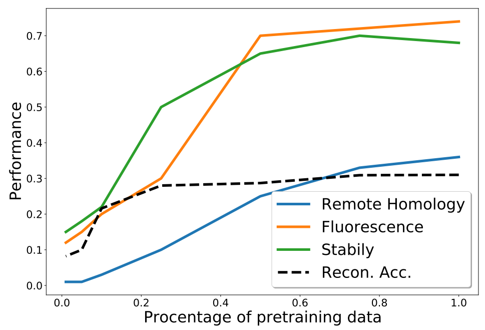

Supplementary Figure 2: Reconstruction and downstream performance as a function of amount of data used during pre-training (in %). Performance refers to Spearman correlation (stability, fluorescence) or accuracy (homology, reconstruction).

reconstruction accuracy flattening out after seeing only 30% of the data, whereas the downstream tasks all increase with more pre-training data. This confirms the discussion from the main paper that the reconstruction accuracy of a model is a poor proxy for the how well the representation will perform on downstream tasks.

Impact of modeling choices on representation

In the main paper, we discuss how learned representations are affected by data preprocessing, choice of modeling architecture and post-hoc dimensionality reduction. It would be convenient to avoid the post-hoc dimensionality reduction step altogether. This is possible for the VAE, by simply setting the latent dimension to 2, but turned out to be infeasible for the sequential models. Even in the case of the bottleneck ResNet, which in many ways is similar to the VAE, it was not possible to train models with a two dimensional representation, which suggests that the low dimensional bottleneck is incompatible with the requirements for the expressivity of the encoder/decoder in these more complex sequential models.

To test the impact of the dimensionality reduction step on the representation, we conducted dimensionality reduction for all sequential models with both PCA and t-SNE (Supplementary Figure 3). Although the t-SNE representations seem to have slightly better separation of phyla locally, the overall patterns produced by the two dimensionality reduction schemes are quite robust, in particular in the most specific representations in the bottom right. We also investigated the impact of dimensionality reduction for the VAE, when trained with a higher dimensional latent space of 30, and applying t-SNE or PCA to reduce it to two dimensions (Supplementary Figure 4). In this case, the PCA seems to

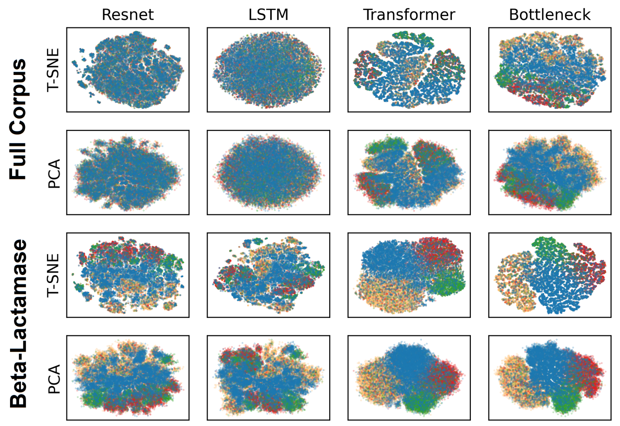

Supplementary Figure 3: Impact of the choice of dimensionality reduction technique on the representation manifolds. This figure corresponds to Figure 2 in the main paper, but includes dimensionality reduction with both PCA and t-SNE.

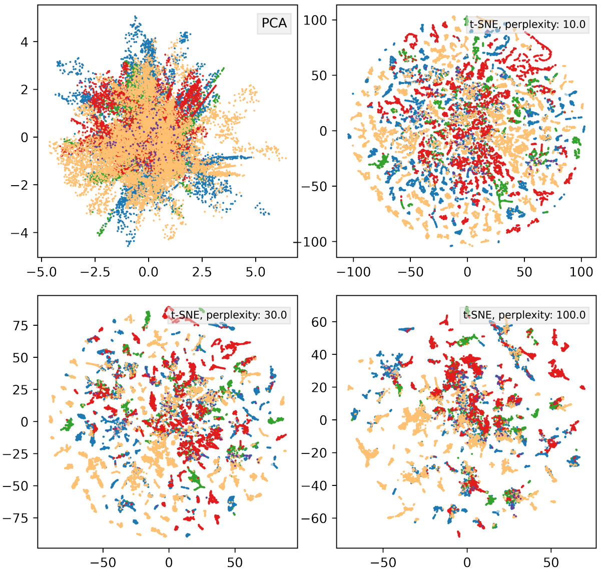

Supplementary Figure 4: Dimensionality reduction on the latent space of a VAE. In contrast to the two dimensional latent space employed in the main paper, we here train a model with 30 latent dimensions. The two plots show the same latent space reduced by either PCA or t-SNE, the latter with different choices of the perplexity parameter.

preserve the star-like structure to some extent, while t-SNE organizes the clusters in a completely different topology, presumably due to the distributional assumptions underlying the t-SNE method.

One aspect of model architecture that was not explored in the main paper was the size of the model, in terms of the number of parameters. The universal, "full-corpus", models explored in Figure 2 in the main paper were all trained on the same dataset with a similar number of parameters. In Supplementary Figure 5 we show the behavior of

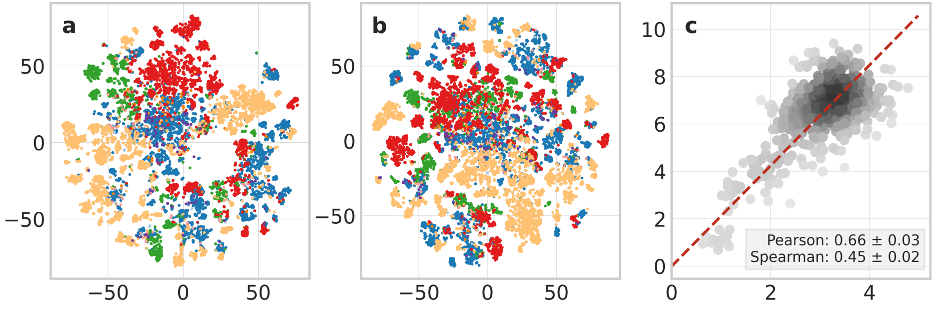

Supplementary Figure 5: Representations extracted from a state-of-the-art large transformer model, ESM-1b [10]. The embeddings were calculated in two different ways: a) extracting sequences from the beta-lactamase multiple sequence alignment used in main paper by removing gaps, b) retrieving the full-length sequences corresponding to the proteins in the alignment. The two approaches lead to comparable representations. Compared to the shallow transformer presented in the main paper, we see that large scale language models capture similar information as obtained from a multiple sequence alignment - at least in terms of correlation to phylogenetic distances (c).

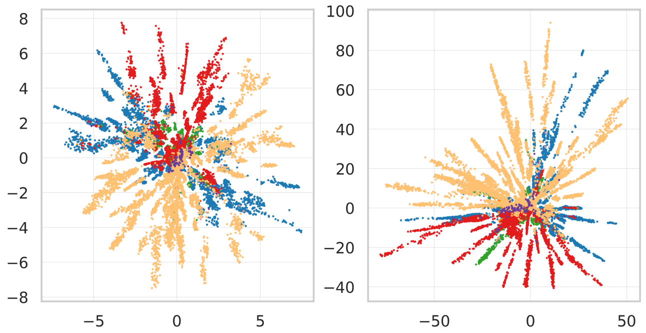

Supplementary Figure 6: Illustration of the difference in obtained representation between a variational autoencoder (left) and a standard autoencoder (right). One of the main differences is that the $\mathcal{N}(0,1)$ prior in the variational autoencoder sets the scale of the latent space.

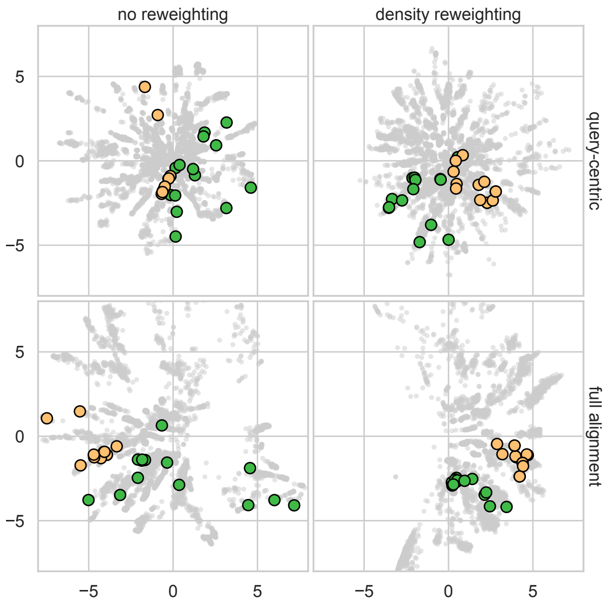

Supplementary Figure 7: The effect of alignment preprocessing on the learned representation. Top row: query-centric alignment where columns are removed if they contain a gap in the query sequence. Bottom row: standard alignment of the same sequences. Left/Right column: whether the sequences are reweighted during training of the model. The green dots correspond to proteins belonging to the same subclass as the query (A1). The yellow dots belong to subclass A2, which is more distant to the query protein.

a state-of-the-art language model, ESM-1b, which has substantially more parameters, and is trained on a much larger dataset [10]. We see that this larger-scale transformer model displays improved separation capabilities compared to models in the top row in Figure 2, and the correlation to phylogenetic distances becomes comparable to that of models trained on the specific family.

While some architecture choices clearly have an impact on the resulting representation, other choices seem to be of little consequence. For instance, we observe that a standard (non-variational) autoencoder produces very similar representations as a variational autoencoder, when trained on the same data set. The inductive bias difference between these two models thus seems to be negligible, apart from the regularizing $\mathcal{N}(0,1)$ prior which sets an overall scale in representation space (Supplementary Figure 6).

Effect of alignment preprocessing

In the main text, we discuss how reweighting of input data and column removal in the alignment can lead to representations with different degrees of selection bias towards a particular query sequence (Figure 6). Visualizations of these four settings are displayed in Supplementary Figure 7.

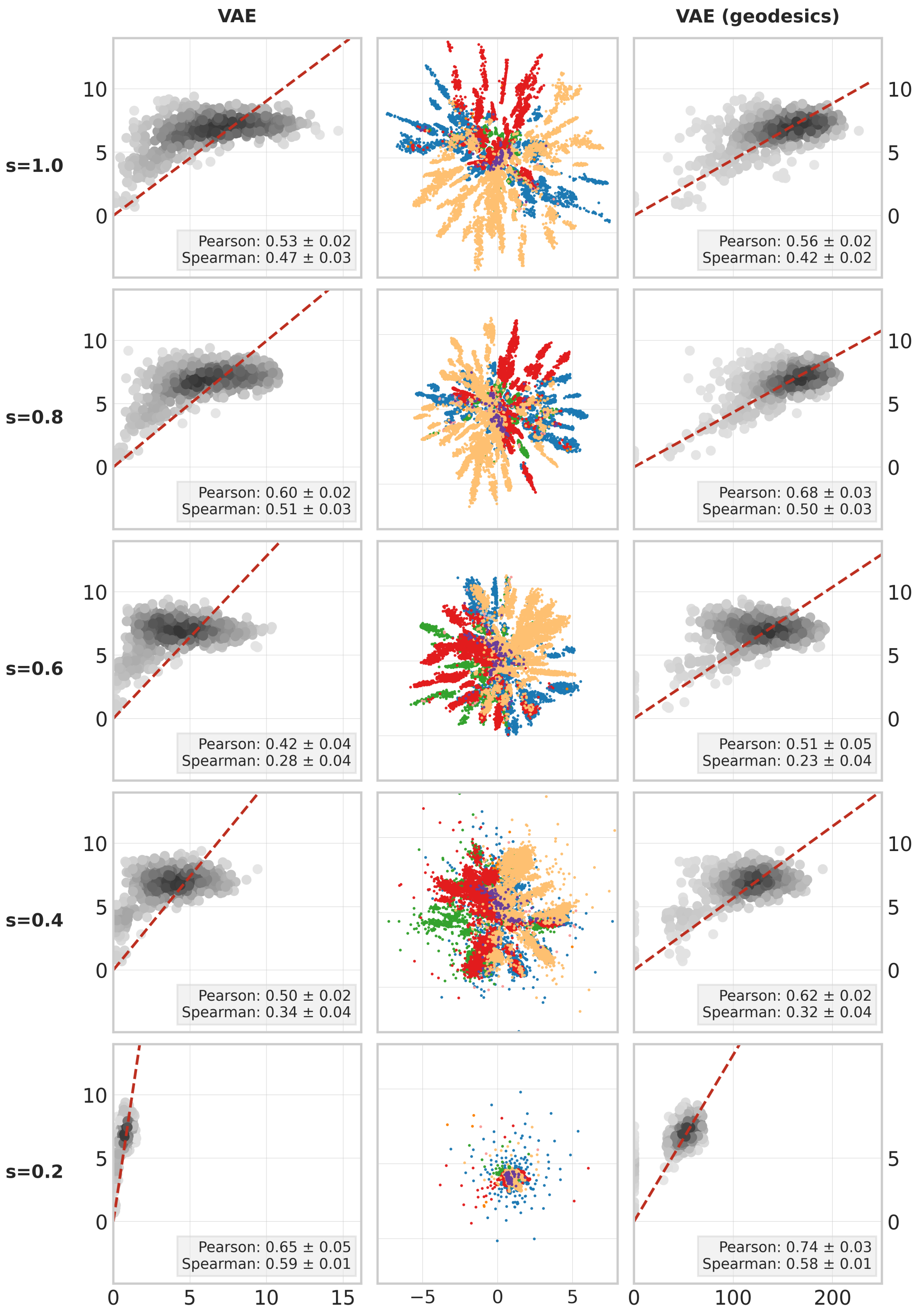

Supplementary Figure 8: The effect of sequence reweighting on the obtained representation. The rows display representations obtained with VAEs trained with different degrees of density reweighting of the input sequences. The s parameter denotes the maximum hamming distance used as a cutoff to define a neighborhood of similar sequences [11]. The $s = 0.8$ setting corresponds to what is referred to as *density reweighting* in the main text and Supplementary Figure 7.

Degree of sequence reweighting

The VAE in Figure 3 in the main paper was trained without conducting sequence reweighting. For completeness, we here include results from training on a dataset where the input sequences are reweighted based on input density, for different choices of the hamming cutoff distance s, which defines how large a neighborhood is considered when estimating the density [11]. As expected, we see a better balanced representation of the different phyla when reweighting, most pronounced in the added weight on the green Firmicutes subtree (Supplementary Figure 8) and the even lower populated phyla that are difficult to discern in the original VAE. The trends in the correlations to phylogenetic distances are similar to those reported in the main paper, again demon-

strating a benefit of employing geodesics rather than euclidean distances.

Impact of initialization on the representation

Although it is commonly accepted that initialization of a neural network has some impact on the resulting models, the ultimate behavior and performance of a model is often fairly robust to different initializations. It is important to stress that this is not the case for learned representations, which can change dramatically depending on the initialization, partly due to the many symmetries in parameter space. As an example, in Supplementary Figure 9 we show the representations of the β-lactamase protein family for 4 different initial seeds. While they all follow the overall tree structure, we see clear variations in the organization of the individual branches of the tree, and we especially observe that the orientation in latent space is arbitrary. This further supports the idea that a Euclidean interpretation of the latent space can be misleading. Figure 4a in the main paper quantifies this effect in terms of the robustness of distances calculations across different training instances.

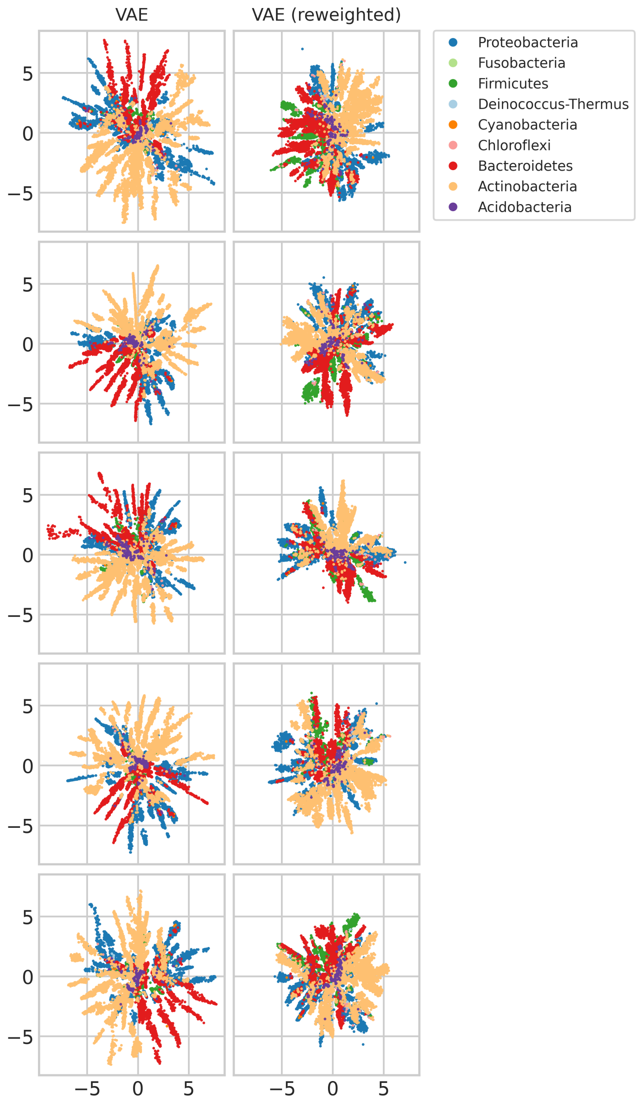

Supplementary Figure 9: Different initializations of the same model. Left column: trained on raw data; right column: trained on density reweighted data ($s = 0.8$). The top left plot corresponds to the model used in the main paper.

Supplementary References

1. El-Gebali, S. *et al.* The Pfam protein families database in 2019. *Nucleic Acids Research* **47,** D427–D432 (2018).
2. Rao, R. *et al.* Evaluating protein transfer learning with TAPE. in *Advances in Neural Information Processing Systems* (2019), 9689–9701.
3. Hou, J., Adhikari, B. & Cheng, J. DeepSF: deep convolutional neural network for mapping protein sequences to folds. *Bioinformatics* **34,** 1295–1303 (2018).
4. Sarkisyan, K. S. *et al.* Local fitness landscape of the green fluorescent protein. *Nature* **533,** 397–401 (2016).
5. Rocklin, G. J. *et al.* Global analysis of protein folding using massively parallel design, synthesis, and testing. *Science* **357,** 168–175 (2017).
6. Armenteros, J. J. A., Johansen, A. R., Winther, O. & Nielsen, H. Language modelling for biological sequences–curated datasets and baselines. *bioRxiv* (2020).
7. The UniProt Consortium. UniProt: a worldwide hub of protein knowledge. *Nucleic Acids Research* **47,** D506–D515 (2018).
8. Riesselman, A. J., Ingraham, J. B. & Marks, D. S. Deep generative models of genetic variation capture the effects of mutations. *Nature Methods* **15,** 816–822 (2018).
9. Devlin, J., Chang, M.-W., Lee, K. & Toutanova, K. BERT: Pre-training of Deep Bidirectional Transformers for Language Understanding. in *Proceedings of the 2019 Conference of the North American Chapter of the Association for Computational Linguistics: Human Language Technologies, Volume 1 (Long and Short Papers)* (2019), 4171–4186.
10. Rives, A. *et al.* Biological structure and function emerge from scaling unsupervised learning

to 250 million protein sequences. *Proceedings of the National Academy of Sciences* **118** (2021).

11. Ekeberg, M., Lövkvist, C., Lan, Y., Weigt, M. & Aurell, E. Improved contact prediction in proteins: Using pseudolikelihoods to infer Potts models. *Physical Review E* **87** (2013).
