# Supplementary material for: Learning meaningful representations of protein sequences
Source: Nat Commun. 2022 Apr 8;13:1914. doi: 10.1038/s41467-022-29443-w (PMC8993921; doi:10.1038/s41467-022-29443-w)
Supplement: Supplementary file 2 — Reporting Summary [file 41467_2022_29443_MOESM2_ESM.pdf]

Corresponding author(s): Wouter Boomsma

Last updated by author(s): Mar 2, 2022

Reporting Summary

Nature Research wishes to improve the reproducibility of the work that we publish. This form provides structure for consistency and transparency in reporting. For further information on Nature Research policies, see our Editorial Policies and the Editorial Policy Checklist.

Statistics

For all statistical analyses, confirm that the following items are present in the figure legend, table legend, main text, or Methods section.

n/a	Confirmed	
☐	☒	The exact sample size (*n*) for each experimental group/condition, given as a discrete number and unit of measurement
☒	☐	A statement on whether measurements were taken from distinct samples or whether the same sample was measured repeatedly
☒	☐	The statistical test(s) used AND whether they are one- or two-sided<br>*Only common tests should be described solely by name; describe more complex techniques in the Methods section.*
☒	☐	A description of all covariates tested
☒	☐	A description of any assumptions or corrections, such as tests of normality and adjustment for multiple comparisons
☐	☒	A full description of the statistical parameters including central tendency (e.g. means) or other basic estimates (e.g. regression coefficient) AND variation (e.g. standard deviation) or associated estimates of uncertainty (e.g. confidence intervals)
☒	☐	For null hypothesis testing, the test statistic (e.g. *F*, *t*, *r*) with confidence intervals, effect sizes, degrees of freedom and *P* value noted<br>*Give P values as exact values whenever suitable.*
☒	☐	For Bayesian analysis, information on the choice of priors and Markov chain Monte Carlo settings
☒	☐	For hierarchical and complex designs, identification of the appropriate level for tests and full reporting of outcomes
☒	☐	Estimates of effect sizes (e.g. Cohen's *d*, Pearson's *r*), indicating how they were calculated

Our web collection on statistics for biologists contains articles on many of the points above.

Software and code

Policy information about availability of computer code

Data collection	The preprocessing scripts are available online under an open source licence: https://github.com/MachineLearningLifeScience/meaningful-protein-representations.
Data analysis	The following software was used in the analysis:<br>Python 3.9.5 using modules: numpy (1.19.5), pytorch (1.9.1), pytorch_lightning (1.2.10), matplotlib (3.4.3), Pandas (1.3.2), Biopython (1.79), Scipy (1.7.0), sklearn (0.24.2), seaborn (0.11.2)<br><br>ClustalO (1.2.3)<br>PAML (4.9j)<br>FastTree (2.1.10)<br><br>The source code and notebooks for all analyses in the the paper are available online under an open source licence: https://github.com/MachineLearningLifeScience/meaningful-protein-representations.

For manuscripts utilizing custom algorithms or software that are central to the research but not yet described in published literature, software must be made available to editors and reviewers. We strongly encourage code deposition in a community repository (e.g. GitHub). See the Nature Research guidelines for submitting code & software for further information.

Data

Policy information about availability of data

All manuscripts must include a data availability statement. This statement should provide the following information, where applicable:

- Accession codes, unique identifiers, or web links for publicly available datasets
- A list of figures that have associated raw data
- A description of any restrictions on data availability

All data used in this manuscript originates from publicly available databases. The sequence data used for pre-training and data for the different protein tasks are available as part of the TAPE repository (https://github.com/songlab-cal/tape). Predefined, curated train/validation/test splits of UniProt were extracted as part of the UniLanguage repository (https://github.com/alrojo/UniLanguage).
Data for the beta-lactamase family was extracted from the Pfam database (\url{https://pfam.xfam.org/family/PF00144}, accessed Jan 2020). Jupyter notebooks are provided which reproduce the figures in the manuscript from this data: https://github.com/MachineLearningLifeScience/meaningful-protein-representations.

Field-specific reporting

Please select the one below that is the best fit for your research. If you are not sure, read the appropriate sections before making your selection.

☒ Life sciences ☐ Behavioural & social sciences ☐ Ecological, evolutionary & environmental sciences

For a reference copy of the document with all sections, see nature.com/documents/nr-reporting-summary-flat.pdf

Life sciences study design

All studies must disclose on these points even when the disclosure is negative.

Sample size	Sample sizes used in our models were determined by availability in the public databases.
Data exclusions	Certain protein sequences were excluded from our analyses when retrieval of additional information from Uniprot failed. This was done once, so all models are affected similarly - and we do not expect this to introduce a bias in the dataset.
Replication	Issues of replication and robustness are discussed explicitly as a topic in the manuscript: Five representation models were trained for each setting to probe the robustness of distance calculations on such representations (Figure 4a). Similarly, 5 random samples of proteins were drawn in Fig 4b, to obtain variances for the Pearson and Spearman correlation coefficients. Statistics of all repetitions were reported.
Randomization	For the supervised learning tasks, train/validation/test splits of the data were predefined by the authors of the benchmark we used. For the repetitions described above, random samples were generated using the random number generator in pytorch, using different seed values.
Blinding	Not applicable: the randomization described above was gone algorithmically and was therefore done without human intervention/selection.

Reporting for specific materials, systems and methods

We require information from authors about some types of materials, experimental systems and methods used in many studies. Here, indicate whether each material, system or method listed is relevant to your study. If you are not sure if a list item applies to your research, read the appropriate section before selecting a response.

Materials & experimental systems

n/a	Involved in the study	
☒	☐	Antibodies
☒	☐	Eukaryotic cell lines
☒	☐	Palaeontology and archaeology
☒	☐	Animals and other organisms
☒	☐	Human research participants
☒	☐	Clinical data
☒	☐	Dual use research of concern

Methods

n/a	Involved in the study	
☒	☐	ChIP-seq
☒	☐	Flow cytometry
☒	☐	MRI-based neuroimaging
