# Supplementary material for: Learning meaningful representations of protein sequences
Source: Nat Commun. 2022 Apr 8;13:1914. doi: 10.1038/s41467-022-29443-w (PMC8993921; doi:10.1038/s41467-022-29443-w)
Supplement: Supplementary file 3 — Peer Review Fil﻿e [file 41467_2022_29443_MOESM3_ESM.pdf]

nature portfolio

Peer Review File

Learning meaningful representations of protein sequences

Open Access This file is licensed under a Creative Commons Attribution 4.0 International License, which permits use, sharing, adaptation, distribution and reproduction in any medium or format, as long as you give appropriate credit to the original author(s) and the source, provide a link to the Creative Commons license, and indicate if changes were made. In the cases where the authors are anonymous, such as is the case for the reports of anonymous peer reviewers, author attribution should be to 'Anonymous Referee' followed by a clear attribution to the source work. The images or other third party material in this file are included in the article's Creative Commons license, unless indicated otherwise in a credit line to the material. If material is not included in the article's Creative Commons license and your intended use is not permitted by statutory regulation or exceeds the permitted use, you will need to obtain permission directly from the copyright holder. To view a copy of this license, visit http://creativecommons.org/licenses/by/4.0/.

Reviewers' Comments:

Reviewer #1:
Remarks to the Author:
The paper studies the criteria for choosing/constructing good protein representations in the context of interpretability (visualization) and transfer learning (training supervised models for different downstream tasks). The authors propose some good practices for extracting and using protein representations.

I believe that the paper addresses many important problems and presents relevant comparison studies that point to interesting results that could be relevant to many future studies using sequence representations from many different unsupervised models recently proposed.

The paper is clearly written with a clear description of a problem statement defined at the beginning of every section and the final conclusion/result presented at the end. However, the methodology part is not always complete and often lacks some details (see below).

The first part of the paper doesn't present any novel methodology, but addresses important questions (pooling strategies for computing global protein representation & introduces criteria for good representation, etc.). Whereas, the second part of the paper is more interesting and presents some novel methodology that can be used in studying geometry of feature spaces for better visualization and interpolation between protein embeddings. Unfortunately, it's very poorly described and hard to follow. Specifically, in the second part of the paper where the authors focus on the geometry of the feature space. Moreover, the proposed metrics can also be used in the context of protein families and functions and not only phylogenetic assignment.
See below for major/minor comments.

Major comments:

Section on computing global representation from local representations: I believe that this is a very important question especially in many studies that are using self-supervised learning (with protein LMs as unsupervised pre-training). However, I believe that this has been explored in many previous studies more extensively than in this manuscript. In ML there is "soft pooling", "K-max pooling", "Max + Avg pooling", etc. For example, see review: Monteiro, J., Alam, M. J., & Falk, T. (2020, May)
Or, a recent paper by https://www.biorxiv.org/content/10.1101/2021.04.25.441334v1.full.pdf
The results presented by the authors are not comprehensive and they cover only major global pooling strategies.

-It's not entirely clear how the authors computed the "Bottleneck" strategy ? I believe some details are missing -- what is the autoencoder (AE) architecture used for computing global representation? What is the input to the AE ?
For the "Concat" strategy, how do you reduce the size of the local representation ? Also, it's not clear how you get global representation to be 2048 (500 x 4 = 2000 ?)

-Instead of visually inspecting the latent spaces (from t-SNE plots in Fig. 2) it would be better if the authors can introduce some quantitative measure of separation between different phyla clusters. For example, average distance between proteins belonging to the same phyla vs average distance between proteins belonging to different phyla.

-Section on geometry of the latent space: the authors introduce a novel distance measure in the

feature space constructed by VAEs and show that the distance has more biological meaning. However, the concept of a curve on latent space is not well defined. What is the parameter t in c(t) ? It's not clear why the authors didn't use notation from VAE when defining energy and geodesics in equation (2)? p(X|Z) instead of f(c) ? c instead of Z ? It's not clear how the authors used the derived equation for energy (equation 3) to compute the shortest path distance in the VAE feature space presented in Fig. 4 (third panel). I believe that it is not clear how this distance is computed in practise. Let's say when we are dealing with the VAE feature space for two sequences with embeddings Z_1 and Z_2 ?

-It would be interesting if the authors can give some explanation why the geometry of the feature spaces from VAEs significantly differs from, for example, the one from the Transformers. Is the emergence of the non-Euclidean space always guaranteed when training models in aligned sequences ?

-The authors focus more on how the distance in feature space describes phylogenetic structure (focusing on a single protein family - beta-lactamases). However, I believe that it would be relevant to see how feature representations from VAE, Transfromer & Resnet correlate with protein family assignments or even functions (GO terms/EC numbers). I would love to see embeddings of proteins belonging to different protein families from Pfam, or having different GO terms/EC numbers. I think this could have interesting applications. For example, can a newly introduced distance measure be used for transferring annotations (Pfam or GO/EC) better than BLAST ?

Minor Comments

-Table 1 doesn indicate what kind of measure is used for comparing different methods ? (accuracy ?)
-Lines 784, 785, 787 & 788: replace D with C in all the sums
-introduce panel labels to Fig. 4 (it's easy to refer to)

Reviewer #2:
Remarks to the Author:
The paper consists of a set of related but somewhat independent analyses in protein representation learning. The authors investigate two settings where representations are used: transfer learning and interpretability. The analyses performed vary significantly in novelty and methodological soundness. Some are quite interesting and provide genuinely new insight, while others are much more straightforward extensions of existing work. My overriding concern with the work is lack of necessary information required to reproduce results and ensure fair comparison between methods - in many cases it is not clear how embeddings are extracted from the model, or what hyper parameters / model architectures are used. I discuss each analysis independently below.

Analysis 1: Constructing a global representation as an average of local representations is suboptimal. In this analysis the authors probe multiple methods for generating a single, global representation of a protein from the local representations provided by standard sequence-to-sequence models from the Rao et al. 2019 TAPE paper. The concatenated and bottleneck embeddings they propose outperform the more commonly used embeddings that they compare to. In particular, the bottleneck embedding significantly outperforms the results from the original TAPE paper.

However, there are significant details omitted from the methodology here. First, it is not stated which base model architecture (Transformer, LSTM, or ResNet) is being used for comparison in Table 1.

Second, the reported results for fluorescence on the attention-based model are significantly worse than those reported in the TAPE paper. This discrepancy may be due to the hyper parameter settings

used; the authors should describe the hyper parameters (batch size, learning rate, etc.) used for fine-tuning. Ideally, the authors should perform separate hyper parameter searches for each embedding type to show them in the best light. If this is computationally unfeasible, the authors should make note of what differentiated their results from the original TAPE paper.

Third, the architecture and training of the bottleneck model is not described. The authors mention on line 165 that this model can be optimized during pre-training, but the paper does not make it clear whether the bottleneck model is optimized during pertaining or only on the downstream supervised task. If it is optimized during pretraining, then are the results from other aggregation methods from the same model or a different model?

Analysis 2: Representations are not immune to overfitting
Here, the authors examine whether fine-tuning model parameters benefits downstream task performance. While it seems to for some tasks and models, it leads to overfitting most of the time. This result does not seem particularly surprising, especially if the difference in performance is evident from the validation performance in all cases (validation performance is not reported in the paper, so it is not possible to determine this). Still, this may be useful for practitioners looking to use this models.

Again the details of training (hyper parameters) are omitted. Additionally, in the previous analysis the authors showed that the aggregation function was important, but then do not note which aggregation function was used for this analysis. Finally, I assumed there should be some correspondence between one of the rows of Table 1 and one of the rows of Table 2, but all numbers seem to be quite different.

Analysis 3: Reconstruction error is not a good measure of representation quality
Here, the authors show that the reconstruction accuracy on the TAPE Pfam validation set does not well predict model performance on a downstream task for the same model using different hyper parameters. Arguably this has been previously shown in the original TAPE paper, where the model with lowest reconstruction error (the Transformer) does not necessarily achieve the highest performance. This analysis extends this to hyper parameters within the same model, and does allow for easier comparison between models.

Of greater note, however, is that the TAPE validation set, which reconstruction error is measured on, is not really designed to be a good estimator of overfitting. While some effort was made to ensure a degree of separation between validation and training set, a proper language modeling validation set here should have much higher sequence identity filtering. As a result, the claim made in this section is too broad; all that can be said is that the TAPE validation set is not a good measure of downstream performance. To make this a general statement, the authors should perform sequence identity clustering and hold out entire clusters at different levels (ideally down to < 30% sequence identity). If such as validation set still cannot detect overfitting, *then* this claim would be reasonable.

Analysis 4: Representation manifolds are shaped by scope, model architecture and data preprocessing
The first set of arguments made here, showing that model representations better separate phyla when trained specifically on a single protein family is well borne out in the t-SNE plots of Figure 2, A-F. The differences between the three models are interesting, especially that the transformer much more clearly separates the phyla.

Training on a single family should significantly increase the speed of overfitting. The authors should describe the training procedure used for the LSTM, Resnet and Transformer on the single family and detail what, if any, steps were taken to avoid this.

A more serious issue is that the t-SNE plots are directly compared to the VAE embedding in Figure 2G. The t-SNE loss tends to produce spherical clusters. The “spiky” nature of the VAE and the clear visual difference between the embeddings are undoubtedly due to the dimensionality reduction used. At best, an argument can be made that the VAE embedding produces sub clusters (which t-SNE *is*

capable of picking up on) that are not present in the t-SNE of the other methods. The analysis should be re-done using more comparable methods, and authors should be wary of directly comparing the outputs of different methods.

Finally, it is unclear how the latent codes for the LSTM, Resnet, and Transformer are generated. As the authors show in analysis 1, this choice can have a significant impact on representations. If a standard mean was used, then perhaps the bottleneck architecture proposed earlier would lead to better global protein latent codes.

Analysis 5: Representation space topology carries relevant information
This analysis, which shows that VAE latent codes correspond reasonably well to phylogenetic clusters, is interesting - though of course the original work showing clustering by phyla is from the Riesselman et al. 2018 DeepSequence paper. It is interesting to see that this clustering is consistent at a more granular level as well, although my suspicion is that this corresponds more closely to a simple hamming-distance measurement. If this is the case, then I suspect that using either the "Concat" or "Bottleneck" methods for generating latent vectors from sequence-to-sequence models may produce similar sub-clusters (I am assuming here that the latent codes for the LSTM, ResNet, and Transformer were generated through a simple mean - if this is not true, the authors should specify the generation method and it may resolve this concern).

Analysis 6: Geometry gives robust representations
This and the following analyses introduce an alternative distance metric to Euclidean distance in the VAE latent space which combines distance in latent with distance in data dimension space. Overall this section is the most interesting, novel, and technically challenging portion of the paper. In addition, extensive information is provided on the implementation of the entropy network, the geodesics, etc. is provided, making this the most reproducible section. (Specifications of model architecture are still not provided, although this is available in the provided code).

The fact that embeddings are robust wrt this particular metric, but not wrt Euclidean distance is quite interesting and gives strong credence to its use.

Analysis 7: Geodesics give meaning to representations
Figure 4 is quite interesting and provides a metric for how closely embeddings match phylogenetic distance, which is useful. As with analysis 5, it would be good to ensure that the transformer latent code is captured via one of the concat/bottleneck methodologies proposed. Figure 5 tries to visually reveal the correspondence between the geodesic distance and phylogenetic distance, but personally I found it a little difficult to interpret. I do not look at phylogenetic tree data very often however, so this may be more interpretable to another reader. One suggestion is to try passing in geodesic distance to an embedding algorithm like t-SNE (many of these allow you to pass in an aribitrary distance matrix - if t-SNE doesn't work, perhaps an alternative would), and "re-embedding" the points such that euclidean distance corresponds to geodesic distance. This may better reveal the clustering visually.

Analysis 8: Data preprocessing affects the geometry
This is a nice experiment that confirms several hypotheses that are common in the protein embedding community. First, that sequence reweighting increases reconstruction accuracy for subclasses the query protein does not belong to, and second that deleting query-sequence gap positions preferentially improves performance around the query protein. In many cases, the goal is to maximize performance around the query protein - but for cases where this is not the goal, this experiment does confirm that using the full sequences alongside sequence reweighting should result in the best embeddings.

Analysis 9: Geodesics provide more meaningful interpolation
This last experiment is perhaps the most interesting. It shows that interpolation along geodesic distance can provide more meaningful interpolates, while direct latent space interpolation may simply

linearly interpolate between the output probability distributions of the endpoints. The authors find that known intermediaries between two sequences fall along the geodesic path. This is very interesting, as there has been significant interest in using VAEs to generate novel protein sequences recently; if interpolation in the latent space is not meaningful it would require the introduction of alternate sampling methodologies (perhaps using the distance definitions provided in this work).

Miscellenious:
Nitpicking slightly, on line 65 the authors state that this paper "starts [the] discussion" of whether learned representations capture meaningful information. Given that this is pretty much exactly the pitch the TAPE paper makes, it seems hard to justify this statement.
In the supplement the authors state the Resnet is trained using next-token-prediction. However, assuming they used the code and model from TAPE for training, the model is trained with masked language modeling.

Reviewer #3:
Remarks to the Author:
The authors explore two aspects of protein sequence "representation" in machine learning: the structure of the internal data when using transfer learning protocols to help train networks on problems with limited datasets, and the visualization of the underlying data.

Transfer learning, they explain, is a process in which machine learning proceeds in two stages. First an unsupervised neural network composed of two halves, an encoder and decoder, is trained to reconstruct its input. Second the newly trained encoder is taken as the first part of another neural network where the second part is then trained in a supervised learning task. By starting with an encoder that is already trained on a large number of unlabeled data points, supervised learning can be performed using a small number of labeled data points. Given how many sequences are available and how few of them will be labeled for particular tasks that biologists might be interested in making predictions for, transfer learning for protein sequences is of high interest to a large number of biologists. Unfortunately, machine learning seems to be as much an art form as it is a science, and so it takes considerable effort to learn the craft. This paper does a really good job of explaining and contrasting the options for network construction and providing a clear set of guidelines when possible about what kinds of networks are most useful for biological data.

There are several natural questions for how to deal with protein sequence data in the context of transfer learning. First: a representation of the entire sequence must be generated, however, proteins are all of different lengths. Typically, scientists will use a recurrent neural network or one based on attention to derive a latent (local) representation of each amino acid in a sequence. How then should these local representations be merged in order to generate a sequence-length independent representation? The authors compared averaging, concatenation, attention, and taking the latent representation from a "bottleneck" auto-encoder and found that the bottleneck approach generated the best results at three separate supervised learning tasks. They then compared whether "fine tuning" during the supervised learning task – i.e. modifying the encoder's parameters learned in the initial unsupervised learning step – improved the three supervised learning tasks and found clear evidence of overfitting. Finally, they examined whether the best meta-parameters of the autoencoders could be determined directly from the reconstruction error. In this case, there was no clear relationship between reconstruction error and how well the supervised learning task would perform; increasing network size improved reconstruction error, but after a point, hurt the supervised learning tasks. I felt the first three results relating to transfer learning were quite strong, and each worthy of individual publication.

The second half of the paper examines what emergent patterns can be found from the autoencoder's representation of the sequences. The authors found that training with LSTM, Resnet, or Transformer

on the (sequence aligned) beta-lactamase protein family that the networks did a decent job at grouping sequences from the same phyla together, as visualized by t-SNE. The Transformer architecture did the best job at this, though, the authors seem to discount the decent jobs done by the other two architectures. The most striking visualization of the data, however, was created by a “VAE,” (which the authors neglect to spell out as a variational auto encoder), as shown in figure 2. (The color scheme in figure 2 makes it appear as if there are only four phyla in the blue, green, yellow, and red colors. If the majority of the data is represented by these four colors, that should be noted in the figure legend.) They note that the VAE generates a tree that reflects a reasonable phylogenetic tree. They do not, however, analyze how a VAE would perform at a transfer learning, or even comment on the idea, which would be useful as figure 2 makes its latent representation seem better. Perhaps the issue is the VAE is trained on only the aligned beta-lactamase sequences and that would make it less useful for transfer learning, or perhaps the issue is that VAE’s are stochastic? Some guidance here would surely be appreciated by this paper’s biologist audience. The authors conclude by talking about representation algebras and note that Euclidean interpretations, while impressive in NLP, define interpolation points that are nonsensical for proteins; geodesics, however, produce sequences that are more meaningful. This part is considerably more technical and perhaps less accessible.

In many ways this paper reads like a review: the datasets and transfer learning tasks are ones taken from other papers, as are the network topologies. Where I think this paper offers the most value is in guiding machine-learning novices in the best ways to construct networks and to understand their limitations. I certainly learned a great deal in the process of reading this paper.

-- Andrew Leaver-Fay

Reviewer #4:
Remarks to the Author:
This manuscript discusses aspects of machine learning models which encode protein-sequence inputs into internal representations. This is a very current and active topic, with new results are rapidly and almost constantly being published. The ideas presented in the paper are at the forefront of research into learning protein sequence datasets.

The analysis is largely divided into two parts, laid out as a series of "implications" for fitting and using these types of models:

One part uses the "TAPE" (ref 5: Rao 2019) dataset/model/suite of "sequential" models, and the authors perform new tests inccuding on the effect of different "global aggregation" strategies, and testing whether fixing the pre-trained model helps prevent overfitting.

The second uses a Variational Autoencoder (VAE), and analyzes the latent space embeddings of the input sequences using Riemannian geodesics (as in Ref 40: Arvanitidis 2018, Ref 48: Kalatzis 2020 and others). This section demonstrates the power of using geodesic distances instead of the latent euclidean distances.

The two types of analysis are presented under a common theme, that of the usefulness and meaningfulness of the "representation" the models make internally of the input protein sequences. This is similar to the discussion in literature of "manifold mismatch" where the question is whether the topology and metric of the latent space matches that of the input space (Davidson et al 2018, Falorsi et al 2018).

One criticism is that while the authors discuss both types of models under a common framework, it is not clear to me that the models have comparable representations. The manuscript sometimes seems like two separate manuscripts: One set of results for sequential models only, and another for VAEs

only. There is a section contrasting the two models, but I have questions about the comparison below. The authors have interesting results in each case separately, however.

Specific questions:

1. Contrasting sequential models with VAE

The authors treat the sequential models and the VAE as comparable and generalize their discussion to make points about both simultaneously. But the two kinds of analysis may be more different than the authors allow.

Tables 1 and 2, figs S1 S2 seem to apply only to the sequential models, while figures 3, 5, 6, 7 apply only to the VAE. The implications at lines 183, 217, 256 seem to only apply to sequential models (the VAE has no "pre-training"), while the implications in lines 368, 469, 499 seem to apply only to the VAE-like models (the sequential models do not have a low-dim embedding, without t-SNE, see below).

As the authors discuss (lines 273-300), sequential models are typically applied to variable-length sequences from multiple protein families, while the VAE models are applied to aligned (equal length) sequences from a single protein family. In other words they typically apply to different kinds of data, though as the authors test the sequential model can also be applied to aligned sequences.

But more importantly, the "latent-space" internal to the VAE seems quite different from the "local representation" of the sequential models. For the sequential models, the local representation is the "next/missing character" internal states, so that there are L (sequence length) separate local representations for each sequence. However, for the VAE model, each sequence is projected into a single 2-dimensional latent space. In other words, the VAE reduces the dimensionality of an input sequence (from L to 2) and embeds the sequence in the low-dim metric space, while the sequential models seem to increase the dimensionality of the representation (from L to L*4 for 4 local dimensions used in the manuscript, before aggregation) and the resulting set-of-local-representations do not explicitly have an interpretation as a metric space.

The discussion contrasting the two models in lines 273-300 is relevant to the field and interesting. The authors conclude that "model architecture [has] a fundamental impact on the resulting representations", which I agree with, but should one then go further and say that the model architectures are different enough their internal representations are not directly comparable, and that only the VAE "embeds" the input into a lower dim space?

Some detailed comments on this distinction:

i. In figure 2, the authors contrast the embedding of the sequential models (panels A-F) with that of the VAE (panel G). But I am not sure A-F are really lower-dim embeddings produced by the sequential models: The dimension reduction to 2 dims was carried out after-the-fact using t-SNE. Is the lack of phylogenetic structure caused by the local-representation mapping, or by the dim reduction of t-SNE? The fact that the VAE latent space looks so different from the t-SNE result perhaps suggests that the latent space of the VAE is a different object conceptually than the local-representation-set of the sequential models. The same applies to Fig 4, left: I think this is based on the distances in the t-SNE 2d plot, so does this result arise because of t-SNE's representation or because of the sequential model representation?

ii. While figure 1 is meant to provide an overview of the methods, it shows only a transformer model with L local representations, and not a VAE. If this diagram were of a VAE, it would have only a single "local representation" which could be used to reconstruct the input. The "task model" and "global-to-local strategies" sections of figure 1 don't seem applicable to the VAE. This was confusing as I initially thought this was meant to show a VAE.

iii. The authors note in footnote 1 that "representation" and "embedding" are used interchangeably in the literature, but should they be? Embedding might be more appropriate for latent spaces with a metric.

iv. In table 1, it was not clearly stated exactly which model is being used. It is a sequential model since the authors imply L local representations in the text, but is it a transformer, LSTM, ResNet? Could the analysis of table 1 be done with the VAE?

2. Comments on the Sequential model analysis

The analysis here (tables 1 and 2) is useful. The results that overtting can be prevented by fixing the model, and that adding an extra learning layer in task output to improves predictions, might not be that surprising though, but perhaps that is because the authors explained the reason for the results so clearly and convincingly.

The methods used to make table 1 need to be explained, there are no methods or code provided for tables 1 or 2. The analysis was done building on the TAPE code, but additional detail could be given on the additions (eg, what is the autoencoder architecture for bottleneck?).

3. Comments on the VAE analysis

Some of the conclusions here do not seem entirely novel, but are new in the sense they are applied specifically in the context of protein sequnces and given context. Eg, fig 7 contrasting the paths in latent space is quite similar to (Ref 40: Arvanitidis 2018, fig 3), who discuss a similar figure saying "induced distances will be large in regions of the latent space where the generator is highly uncertain, these paths paths and distances. will then tend to follow the data in the latent space" (compare to line 607). Similarly, ref 40 says "In general, latent space distances [...] are sensitive to specifics of the underlying neural nets. It is therefore more robust to consider infinitesimal distances along the data manifold in the input space. [i.e, geodesics]" (compare to line 469 on robustness).

Some other comments on the VAE:

Are figures 3 and 5 made using sequences weighted to account for density in sequence space using a sequence similarity cutoff? If such weighting is performed, it might erase much of the phylgenetic structure, which could lead to quite a different picture and to different geodesics. It would be interesting to test this using the reweighted data used in fig 6, in comparison.

The results in fig 7 showing that the "unsupported" regions latent space (as in points 3,4,5 in the euclidean path in fig 7) yields high-entropy decodings has long raised questions to me about how VAEs behave. The VAE prior (a gaussian) suggests, perhaps, that these regions should correspond to valid sequences, and they should be sampled during the training/decoding process. Similarly, during the training process, why does the VAE not learn to map the data to a smooth gausian (the prior) in latent space, as should happen if the encoder properly maps to the prior? I am not sure if it is fair to ask the authors to explain these questions since they are deep questions about VAEs, but if the authors have any insights they would be good additions to the manuscript.

Is it possible that there is some circularity in the results, eg of figure 3? The geodesics are constructed based on distances in the one-hot space, so should it be surprising that the geodesics match the phylogenetic distances well, since the phylogneetic distances are also based on distances in the one-hot space? Likewise this would explain why the geodesic distances are resilient to reparametrizations of the latent space.

Point-by-point response

We thank the reviewers for their thorough comments and suggestions. Below is a point-by-point response to each of the issues raised.

Reviewer #1 (Expertise: DL, protein design)

> *The paper studies the criteria for choosing/constructing good protein representations in the context of interpretability (visualization) and transfer learning (training supervised models for different downstream tasks). The authors propose some good practices for extracting and using protein representations.*
>
> *I believe that the paper addresses many important problems and presents relevant comparison studies that point to interesting results that could be relevant to many future studies using sequence representations from many different unsupervised models recently proposed.*
>
> *The paper is clearly written with a clear description of a problem statement defined at the beginning of every section and the final conclusion/result presented at the end.*

We thank the reviewer for his/her positive comments.

> *However, the methodology part is not always complete and often lacks some details (see below).*
>
> *The first part of the paper doesn't present any novel methodology, but addresses important questions (pooling strategies for computing global protein representation & introduces criteria for good representation, etc.). Whereas, the second part of the paper is more interesting and presents some novel methodology that can be used in studying geometry of feature spaces for better visualization and interpolation between protein embeddings. Unfortunately, it's very poorly described and hard to follow. Specifically, in the second part of the paper where the authors focus on the geometry of the feature space. Moreover, the proposed metrics can also be used in the context of protein families and functions and not only phylogenetic assignment. See below for major/minor comments.*

Upon rereading our manuscript, we acknowledge that some of the methodological parts were not clearly explained. In the new version of the manuscript, we have clarified these descriptions, and added additional information to the supporting material such that all methods are fully explained. Finally, code for all experiments are now provided as part of the paper, with the aim of making it fully reproducible. See detailed comments below.

> *Major comments:*

Section on computing global representation from local representations: I believe that this is a very important question especially in many studies that are using self-supervised learning (with protein LMs as unsupervised pre-training). However, I believe that this has been explored in many previous studies more extensively than in this manuscript. In ML there is "soft pooling", "K-max pooling", "Max + Avg pooling", etc. For example, see review: Monteiro, J., Alam, M. J., & Falk, T. (2020, May)
Or, a recent paper by
https://www.biorxiv.org/content/10.1101/2021.04.25.441334v1.full.pdf
The results presented by the authors are not comprehensive and they cover only major global pooling strategies.

We thanks the reviewer for pointing us to these references and acknowledge that our section on sequence aggregation was not sufficiently comprehensive. In our revision of the paper, we have added comparisons to methods: K-max, Max + Avg and Light Attention as described in the "Light Attention Predicts Protein Location from the Language of Life" paper and cited the relevant literature. These new results are consistent with the conclusion drawn in the previous version of the paper, that learning global representations are better than obtaining them post-hoc from local representations.

-It's not entirely clear how the authors computed the "Bottleneck" strategy ? I believe some details are missing -- what is the autoencoder (AE) architecture used for computing global representation? What is the input to the AE ?
For the "Concat" strategy, how do you reduce the size of the local representation ? Also, it's not clear how you get global representation to be 2048 (500 x 4 = 2000 ?)

We agree that this was not sufficiently clear. In the new version of the manuscript we now describe the architectures used in all experiments in supplementary material (see "Bottleneck AutoEncoder" section in particular), and now also provide access to all our code so that any further missing details can be clarified that way. For the "Concat" strategy we incorrectly wrote that we set the maximal sequence length to 500 where it should have been 512. Multipling by 4 we arrive at same latent size as the other models, 2048. We have corrected this in the main paper.

-Instead of visually inspecting the latent spaces (from t-SNE plots in Fig. 2) it would be better if the authors can introduce some quantitative measure of separation between different phyla clusters. For example, average distance between proteins belonging to the same phyla vs average distance between proteins belonging to different phyla.

We agree that quantitative experiments should generally be preferred, and when planning the experiments, we discussed at length how to do properly do these comparisons. Since one of the points later in the paper is that it is potentially misleading to calculate Euclidean distances in latent space, we were reluctant to use distances as the basis for as assessment of quality of a representation in this earlier part of the paper - instead opting to focus on the qualitative differences as they appear visually. However, in the sections following it, we then focus on a quantitative description through distance calculations (Fig 4b), and compare how distances in latent space correlate to a "ground truth" (phylogenetic distances). As far as we

can see, the correlation to phylogenetic distances is fairly similar to what is proposed by the reviewer (distances within phyla being smaller than distances between phyla). We have now also added a figure to quantitatively demonstrate the robustness of these distances upon retraining the representation model (Fig 4a).

> *-Section on geometry of the latent space: the authors introduce a novel distance measure in the feature space constructed by VAEs and show that the distance has more biological meaning.*
> *However, the concept of a curve on latent space is not well defined. What is the parameter t in c(t) ? It's not clear why the authors didn't use notation from VAE when defining energy and geodesics in equation (2)? p(X|Z) instead of f(c) ? c instead of Z ? It's not clear how the authors used the derived equation for energy (equation 3) to compute the shortest path distance in the VAE feature space presented in Fig. 4 (third panel). I believe that it is not clear how this distance is computed in practise. Let's say when we are dealing with the VAE feature space for two sequences with embeddings Z_1 and Z_2 ?*

We acknowledge that this was not sufficiently clear in the original manuscript. We have now made the notation more consistent, explained the background for the energy equation, and described in greater detail how the computation is done in practice (Methods -> Optimizing geodesics). In addition, we have greatly expanded our notebooks to show examples of how these distance calculations are done in practice, which should be of help to anyone who wishes to reproduce the calculation.

> *-It would be interesting if the authors can give some explanation why the geometry of the feature spaces from VAEs significantly differs from, for example, the one from the Transformers. Is the emergence of the non-Euclidean space always guaranteed when training models in aligned sequences ?*

This is an interesting point. The deviation from a Euclidean geometry can be defined for the VAE because we have a smooth decoder that maps from a latent space of fixed dimension to an observation space of fixed dimension. This is the key reason for the importance of using aligned sequences of fixed length. In principle any model that smoothly decodes to a fixed dimensional space could be given a Riemannian interpretation. Here it is worth noting that we have found the uncertainty of the decoder to be crucial for getting a useful geometry, so probabilistic decoders are to be preferred even if such is not a mathematical requirement. In our new version of the manuscript we have added a paragraph discussing this issue (in "Analysing representations appropriately").

> *The authors focus more on how the distance in feature space describes phylogenetic structure (focusing on a single protein family - beta-lactamases). However, I believe that it would be relevant to see how feature representations from VAE, Transfromer & Resnet correlate with protein family assignments or even functions (GO terms/EC numbers). I would love to see embeddings of proteins belonging to different protein families from Pfam, or having different GO terms/EC numbers. I think this could have interesting applications. For example, can a newly introduced distance measure be used for transferring annotations (Pfam or GO/EC) better than BLAST ?*

We agree that this would be extremely interesting to pursue. The problem is that currently, the VAE requires a multiple sequence alignment as input, and can therefore not easily be expanded to much more distant phylogenetic relationships. We did take a look at more fine-grained phylogenetic assignments, such as subfamilies, which we explore in the section on how subclass A1 and A2 are separated in latent space (fig 7). We have an ongoing project in the lab investigating the correlation of latent space position to functional traits and experimental observations such as the protein stability, but this turns out to be multifaceted story, which we found did not fit into the scope of the current paper. The idea of using latent spaces to transfer annotations and support BLAST-like searches is highly relevant. The latter was explored in a recent paper by Morton et al (https://doi.org/10.1101/2020.11.03.365932), and such analyses might indeed be improved by better treatment of the underlying geometry. We have now added a paragraph in the discussion (under "Analysing representations appropriately") mentioning these potential applications, which we hope to explore further in the near future. We thanks the reviewer for this suggestion.

> *Minor Comments*
> *-Table 1 doesn indicate what kind of measure is used for comparing different methods ? (accuracy ?)*
> *-Lines 784, 785, 787 & 788: replace D with C in all the sums*
> *-introduce panel labels to Fig. 4 (it's easy to refer to)*

Thanks for pointing out these minor issues. The suggestions have been incorporated in the new version of the manuscript.

Reviewer #2 (Expertise: DL, protein design):

> *The paper consists of a set of related but somewhat independent analyses in protein representation learning. The authors investigate two settings where representations are used: transfer learning and interpretability. The analyses performed vary significantly in novelty and methodological soundness. Some are quite interesting and provide genuinely new insight, while others are much more straightforward extensions of existing work. My overriding concern with the work is lack of necessary information required to reproduce results and ensure fair comparison between methods - in many cases it is not clear how embeddings are extracted from the model, or what hyper parameters / model architectures are used. I discuss each analysis independently below.*

We thank the reviewer for his careful reading of our manuscript, and address each of the raised concerns below.

> *Analysis 1: Constructing a global representation as an average of local representations is suboptimal.*
> *In this analysis the authors probe multiple methods for generating a single, global representation of a protein from the local representations provided by standard sequence-to-sequence models from the Rao et al. 2019 TAPE paper. The*

concatenated and bottleneck embeddings they propose outperform the more commonly used embeddings that they compare to. In particular, the bottleneck embedding significantly outperforms the results from the original TAPE paper.

However, there are significant details omitted from the methodology here. First, it is not stated which base model architecture (Transformer, LSTM, or ResNet) is being used for comparison in Table 1.

We acknowledge that we in our original manuscript did not describe the model architectures in enough detail. In our revision of the paper, we now clearly state the model architectures for each experiment in the Supporting Information, and provide code to reproduce each of the experiments. With respect to the specific question about Table 1, the caption now states that the base model is a Resnet for all entries in the table.

Second, the reported results for fluorescence on the attention-based model are significantly worse than those reported in the TAPE paper. This discrepancy may be due to the hyper parameter settings used; the authors should describe the hyper parameters (batch size, learning rate, etc.) used for fine-tuning. Ideally, the authors should perform separate hyper parameter searches for each embedding type to show them in the best light. If this is computationally unfeasible, the authors should make note of what differentiated their results from the original TAPE paper.

We thank the reviewer for bringing up this point, which is indeed important to clarify. The reason for the discrepancy was that we in Table 2 (previously Table 1) used a Resnet model as the common backbone for all models, and that we use a no-fine-tuning strategy (since this follows from our conclusion in the first experiment). The combination of these choices happens to lead to a rather low score for the Fluorescence task. We use exactly the same hyperparameter settings as in the TAPE paper, as the tasks are in principle identical and only the aggregation strategy is changing. This story line has become substantially clearer in the new version of the manuscript, where we have reordered the first two experiments so that we now start by considering the different model choices (Resnet, LSTM, Transformer) with choices of fine-tuning/no fine-tuning, and then proceed with one such choice (Resnet, no fine-tuning) for the subsequent experiments.

Third, the architecture and training of the bottleneck model is not described. The authors mention on line 165 that this model can be optimized during pre-training, but the paper does not make it clear whether the bottleneck model is optimized during pertaining or only on the downstream supervised task. If it is optimized during pretraining, then are the results from other aggregation methods from the same model or a different model?

We agree that this was not sufficiently clearly described. The bottleneck is an integrated part of the embedding model, and is thus trained during pretraining, while the other methods are calculated posthoc, and can therefore be calculated on the same previously trained model. All models in the table are based on a Resnet architecture. We now state all this explicitly in the caption for Table 2 (previously Table 1).

Analysis 2: Representations are not immune to overfitting

Here, the authors examine whether fine-tuning model parameters benefits downstream task performance. While it seems to for some tasks and models, it leads to overfitting most of the time. This result does not seem particularly surprising, especially if the difference in performance is evident from the validation performance in all cases (validation performance is not reported in the paper, so it is not possible to determine this). Still, this may be useful for practitioners looking to use this models.

Again the details of training (hyper parameters) are omitted. Additionally, in the previous analysis the authors showed that the aggregation function was important, but then do not note which aggregation function was used for this analysis. Finally, I assumed there should be some correspondence between one of the rows of Table 1 and one of the rows of Table 2, but all numbers seem to be quite different.

In the new version of the manuscript, we now specify which model architecture is used in each section (with details provided in supporting material). We have reorganized the sections so that this is now the first experiment in the paper, and have changed the focus to specifically discuss the practice of fine-tuning (rather than the broader question of overfitting), under the title "Fine-tuning can be detrimental to performance". We therefore now discuss these results without mentioning the Bottleneck strategy (which is introduced subsequently in experiment 2), and instead use the attention-based aggregation function for all experiments, as this seems to the the default choice in the literature. Regarding the correspondence between Table 1 and Table 2, we point out that “Pre+Fix” row for resnet based architecture in Table 1 (previously Table 2) corresponds exactly to the “Attention” row in Table 2 (previously Table 1). However, we acknowledge that this was difficult to spot in the previous version of the manuscript.

Analysis 3: Reconstruction error is not a good measure of representation quality
Here, the authors show that the reconstruction accuracy on the TAPE Pfam validation set does not well predict model performance on a downstream task for the same model using different hyper parameters. Arguably this has been previously shown in the original TAPE paper, where the model with lowest reconstruction error (the Transformer) does not necessarily achieve the highest performance. This analysis extends this to hyper parameters within the same model, and does allow for easier comparison between models.

*Of greater note, however, is that the TAPE validation set, which reconstruction error is measured on, is not really designed to be a good estimator of overfitting. While some effort was made to ensure a degree of separation between validation and training set, a proper language modeling validation set here should have much higher sequence identity filtering. As a result, the claim made in this section is too broad; all that can be said is that the TAPE validation set is not a good measure of downstream performance. To make this a general statement, the authors should perform sequence identity clustering and hold out entire clusters at different levels (ideally down to < 30% sequence identity). If such as validation set still cannot detect overfitting, *then* this claim would be reasonable.*

It was not our intention to overshadow earlier results from the literature. We have now added a sentence to the manuscript acknowledging that our finding is consistent with what was reported in the original TAPE article.
We thank the reviewer for pointing out a potential flaw in our conclusion based on reconstruction error. As suggested, we have now redone the experiment, retraining all models using the carefully constructed partition of Uniprot constructed by Armenteros et al to avoid overlap between splits. The new results turned out to be consistent with those reported in the previous version.

> Analysis 4: Representation manifolds are shaped by scope, model architecture and data preprocessing
> The first set of arguments made here, showing that model representations better separate phyla when trained specifically on a single protein family is well borne out in the t-SNE plots of Figure 2, A-F. The differences between the three models are interesting, especially that the transformer much more clearly separates the phyla.
>
> Training on a single family should significantly increase the speed of overfitting. The authors should describe the training procedure used for the LSTM, Resnet and Transformer on the single family and detail what, if any, steps were taken to avoid this.

These are indeed important points. We have added a paragraph in supplementary material on how the models were trained on the beta-lactamase familie to prevent overfitting.

> A more serious issue is that the t-SNE plots are directly compared to the VAE embedding in Figure 2G. The t-SNE loss tends to produce spherical clusters. The “spiky” nature of the VAE and the clear visual difference between the embeddings are undoubtedly due to the dimensionality reduction used. At best, an argument can be made that the VAE embedding produces sub clusters (which t-SNE *is* capable of picking up on) that are not present in the t-SNE of the other methods. The analysis should be re-done using more comparable methods, and authors should be wary of directly comparing the outputs of different methods.

The comparison is indeed difficult to do properly, but in a way, this is the point of the figure: Practitioners use very different methods to obtain representations, and it is often not clear which impact it has on the resulting representation. Our point was that we must work towards establishing ways to discuss the quality of representations, even when they are obtained using incomparable methods. We do, however, agree on the need to separate between *learned* representations and *post-hoc* dimensionality reduction. We now explicitly address the role of the dimensionality reduction step, by including a version of Figure 2 in supporting material where we replace the t-SNE dimensionality reduction with simple PCA, and also retrain a VAE with higher dimensional latent space, to investigate how dimensionality reductions like PCA and t-SNE can alter the star-like pattern we observe when training a VAE with a latent dimension of two (Fig S3 and S4).

> *Finally, it is unclear how the latent codes for the LSTM, Resnet, and Transformer are generated. As the authors show in analysis 1, this choice can have a significant impact on representations. If a standard mean was used, then perhaps the*

bottleneck architecture proposed earlier would lead to better global protein latent codes.

As described above, we have now clarified this in the paper. In the table in question (which is now Table 1), we use the attention based aggregation function, which seems to be the default choice in the litterature. In the new version of the manuscript, we now introduce the Bottleneck in the second experiment, as an improvement of the base Resnet architecture in the first experiment. It is, however, true that we do not revisit the other model architectures in combination with the Bottleneck strategy. The convolutional structure of the Resnet naturally lent itself to the required down and upsampling operations, while this is less straightforward in the context of an LSTM or a Transformer (see comment below for further technical details). Although we agree that it could be highly fruitful to pursue this direction (we have added this point to the Discussion), we felt that developing such a model was outside the scope of the current work.

Analysis 5: Representation space topology carries relevant information
This analysis, which shows that VAE latent codes correspond reasonably well to phylogenetic clusters, is interesting - though of course the original work showing clustering by phyla is from the Riesselman et al. 2018 DeepSequence paper. It is interesting to see that this clustering is consistent at a more granular level as well, although my suspicion is that this corresponds more closely to a simple hamming-distance measurement. If this is the case, then I suspect that using either the "Concat" or "Bottleneck" methods for generating latent vectors from sequence-to-sequence models may produce similar sub-clusters (I am assuming here that the latent codes for the LSTM, ResNet, and Transformer were generated through a simple mean - if this is not true, the authors should specify the generation method and it may resolve this concern).

The latent codes for the LSTM, ResNet and Transformer were indeed generated using a simple mean, which we now state explicitly in the caption of Figure 2. We thank the reviewer for the suggestion of adding a plot with the Bottleneck strategy to the figure as well, which we have now included. The representations from the Bottleneck strategy turn out to be qualitatively similar to those of the Transformer.

Analysis 6: Geometry gives robust representations
This and the following analyses introduce an alternative distance metric to Euclidean distance in the VAE latent space which combines distance in latent with distance in data dimension space. Overall this section is the most interesting, novel, and technically challenging portion of the paper. In addition, extensive information is provided on the implementation of the entropy network, the geodesics, etc. is provided, making this the most reproducible section. (Specifications of model architecture are still not provided, although this is available in the provided code).

The fact that embeddings are robust wrt this particular metric, but not wrt Euclidean distance is quite interesting and gives strong credence to its use.

We thank the reviewer for the positive remarks. Our new version of the manuscript now contains model specifications for all parts of the manuscript in the Supporting Information.

Additionally, we have updated our code repository to contain the exact implementation for all experiments, and have added a jupyter notebook covering the experiments on the correlation between phylogenetic distances and geodesics.

> *Analysis 7: Geodesics give meaning to representations*
> *Figure 4 is quite interesting and provides a metric for how closely embeddings match phylogenetic distance, which is useful. As with analysis 5, it would be good to ensure that the transformer latent code is captured via one of the concat/bottleneck methodologies proposed. Figure 5 tries to visually reveal the correspondence between the geodesic distance and phylogenetic distance, but personally I found it a little difficult to interpret. I do not look at phylogenetic tree data very often however, so this may be more interpretable to another reader. One suggestion is to try passing in geodesic distance to an embedding algorithm like t-SNE (many of these allow you to pass in an aribitrary distance matrix - if t-SNE doesn't work, perhaps an alternative would), and "re-embedding" the points such that euclidean distance corresponds to geodesic distance. This may better reveal the clustering visually.*

As described above, we do not have a model combining the Transformer architecture with the Bottleneck or Concatenation aggregation strategies. We experimented with this briefly, but in our hands, the transformer required such a high dimensional internal representation for training, that both the Bottleneck and Concatenation strategies became unfeasibly demanding in terms of computational and memory needs. We are confident that such a model could be developed if enough time was invested, but leave this for future work. Regarding the suggestion of using geodesic distances as input to t-SNE (or similar methods), then it should be noted that we only compute these on the VAE representation, which is already two-dimensional. A t-SNE visualization would, thus, have to be dimensionality preserving. We did experiment with such plots using t-SNE, MDS and Isomap, but did not find the resulting plots to be informative.

> *Analysis 8: Data preprocessing affects the geometry*
> *This is a nice experiment that confirms several hypotheses that are common in the protein embedding community. First, that sequence reweighting increases reconstruction accuracy for subclasses the query protein does not belong to, and second that deleting query-sequence gap positions preferentially improves performance around the query protein. In many cases, the goal is to maximize performance around the query protein - but for cases where this is not the goal, this experiment does confirm that using the full sequences alongside sequence reweighting should result in the best embeddings.*

> *Analysis 9: Geodesics provide more meaningful interpolation*
> *This last experiment is perhaps the most interesting. It shows that interpolation along geodesic distance can provide more meaningful interpolates, while direct latent space interpolation may simply linearly interpolate between the output probability distributions of the endpoints. The authors find that known intermediaries between two sequences fall along the geodesic path. This is very interesting, as there has been significant interest in using VAEs to generate novel protein sequences recently; if interpolation in the latent space is not meaningful it would require the introduction of*

alternate sampling methodologies (perhaps using the distance definitions provided in this work).

We thank the reviewer for the positive remarks.

Miscellenious:
Nitpicking slightly, on line 65 the authors state that this paper “starts [the] discussion” of whether learned representations capture meaningful information. Given that this is pretty much exactly the pitch the TAPE paper makes, it seems hard to justify this statement.

This was thoughtless of us. We have rephrased the sentence.

In the supplement the authors state the Resnet is trained using next-token-prediction. However, assuming they used the code and model from TAPE for training, the model is trained with masked language modeling.

Indeed. This has now been corrected.

Reviewer #3 (Expertise: protein design):

The authors explore two aspects of protein sequence “representation” in machine learning: the structure of the internal data when using transfer learning protocols to help train networks on problems with limited datasets, and the visualization of the underlying data.

Transfer learning, they explain, is a process in which machine learning proceeds in two stages. First an unsupervised neural network composed of two halves, an encoder and decoder, is trained to reconstruct its input. Second the newly trained encoder is taken as the first part of another neural network where the second part is then trained in a supervised learning task. By starting with an encoder that is already trained on a large number of unlabeled data points, supervised learning can be performed using a small number of labeled data points. Given how many sequences are available and how few of them will be labeled for particular tasks that biologists might be interested in making predictions for, transfer learning for protein sequences is of high interest to a large number of biologists. Unfortunately, machine learning seems to be as much an art form as it is a science, and so it takes considerable effort to learn the craft. This paper does a really good job of explaining and contrasting the options for network construction and providing a clear set of guidelines when possible about what kinds of networks are most useful for biological data.

There are several natural questions for how to deal with protein sequence data in the context of transfer learning. First: a representation of the entire sequence must be generated, however, proteins are all of different lengths. Typically, scientists will use a recurrent neural network or one based on attention to derive a latent (local)

> *representation of each amino acid in a sequence. How then should these local representations be merged in order to generate a sequence-length independent representation? The authors compared averaging, concatenation, attention, and taking the latent representation from a "bottleneck" auto-encoder and found that the bottleneck approach generated the best results at three separate supervised learning tasks. They then compared whether "fine tuning" during the supervised learning task – i.e. modifying the encoder's parameters learned in the initial unsupervised learning step – improved the three supervised learning tasks and found clear evidence of overfitting. Finally, they examined whether the best meta-parameters of the autoencoders could be determined directly from the reconstruction error. In this case, there was no clear relationship between reconstruction error and how well the supervised learning task would perform; increasing network size improved reconstruction error, but after a point, hurt the supervised learning tasks. I felt the first three results relating to transfer learning were quite strong, and each worthy of individual publication.*

We thank the reviewer for the positive remarks.

> *The second half of the paper examines what emergent patterns can be found from the autoencoder's representation of the sequences. The authors found that training with LSTM, Resnet, or Transformer on the (sequence aligned) beta-lactamase protein family that the networks did a decent job at grouping sequences from the same phyla together, as visualized by t-SNE. The Transformer architecture did the best job at this, though, the authors seem to discount the decent jobs done by the other two architectures.*

We did not intend to discount the other architectures. The intention behind the figure was primarily to illustrate that different architectures give rise to very different representations of the same data - and that these might be more or less appropriate for a given task at hand. In our new version of the manuscript, we have rephrased this to make it clearer. We have also added another column to the figure, demonstrating that the combination of Resnet and Bottleneck aggregation is on par with the Transformer setting. Finally, we have in supporting material included a figure showing how these representations are effected by switching to a different dimensionality reduction technique (Fig S3-S4).

> The most striking visualization of the data, however, was created by a "VAE," (which the authors neglect to spell out as a variational auto encoder), as shown in figure 2. (The color scheme in figure 2 makes it appear as if there are only four phyla in the blue, green, yellow, and red colors. If the majority of the data is represented by these four colors, that should be noted in the figure legend.)

We thank the reviewer for pointing out the missing abbreviation. We now write out Variational Autoencoder the first time it is mentioned.
Indeed, *Actinobacteria*, *Bacteroidetes*, *Firmicutes* and *Proteobacteria* have higher populations and dominate the visualization. In supporting information, we have now included a visualization for a VAE trained on a dataset where sequences are reweighted based on input density, where you can see the other phyla appear more clearly (Fig S5). We have

added this observation to the SI text as well (Under "VAE trained on reweighted sequence data").

> They note that the VAE generates a tree that reflects a reasonable phylogenetic tree. They do not, however, analyze how a VAE would perform at a transfer learning, or even comment on the idea, which would be useful as figure 2 makes its latent representation seem better. Perhaps the issue is the VAE is trained on only the aligned beta-lactamase sequences and that would make it less useful for transfer learning, or perhaps the issue is that VAE's are stochastic? Some guidance here would surely be appreciated by this paper's biologist audience.

This is a very good point. Indeed, as you write, the VAE is trained on sequences from a single family, while the three transfer-learning tasks we consider in the first part of the paper cover many different proteins. This means we cannot directly test the VAE approach in that same setting. It is, however, certainly possible to use a VAE in a transfer learning setting, by learning a representation on all proteins in a single protein family, and subsequently use this as the basis for a downstream model that optimizes a particular trait of interest. We have added a paragraph to the paper to make this point clearer (under "Representations for transfer learning")

> The authors conclude by talking about representation algebras and note that Euclidean interpretations, while impressive in NLP, define interpolation points that are nonsensical for proteins; geodesics, however, produce sequences that are more meaningful. This part is considerably more technical and perhaps less accessible.

In an attempt to make the last part more accessible, we now have rephrased parts of it, and made the notation more consistent.

> In many ways this paper reads like a review: the datasets and transfer learning tasks are ones taken from other papers, as are the network topologies. Where I think this paper offers the most value is in guiding machine-learning novices in the best ways to construct networks and to understand their limitations. I certainly learned a great deal in the process of reading this paper.

Based on comments from the 4 reviewers, it seems that different parts of the paper provide value to different parts of the audience, which was also our intention. The initial part is indeed partially a review (although it includes some new models designs and insights as well) which is perhaps most relevant to biological readers entering the field, while the latter part contains novel methodological contributions which will be most interesting for readers who are more familiar to the field.

Reviewer #4 (Expertise: DL, protein design):

> *This manuscript discusses aspects of machine learning models which encode protein-sequence inputs into internal representations. This is a very current and active topic, with new results are rapidly and almost constantly being published. The*

ideas presented in the paper are at the forefront of research into learning protein sequence datasets.

The analysis is largely divided into two parts, laid out as a series of "implications" for fitting and using these types of models:

One part uses the "TAPE" (ref 5: Rao 2019) dataset/model/suite of "sequential" models, and the authors perform new tests inccuding on the effect of different "global aggregation" strategies, and testing whether fixing the pre-trained model helps prevent overfitting.

The second uses a Variational Autoencoder (VAE), and analyzes the latent space embeddings of the input sequences using Riemannian geodesics (as in Ref 40: Arvanitidis 2018, Ref 48: Kalatzis 2020 and others). This section demonstrates the power of using geodesic distances instead of the latent euclidean distances.

The two types of analysis are presented under a common theme, that of the usefulness and meaningfulness of the "representation" the models make internally of the input protein sequences. This is similar to the discussion in literature of "manifold mismatch" where the question is whether the topology and metric of the latent space matches that of the input space (Davidson et al 2018, Falorsi et al 2018).

We thank the reviewer for pointing out the connection to manifold mismatch. We have added a sentence about this to the paper, citing the relevant work.

One criticism is that while the authors discuss both types of models under a common framework, it is not clear to me that the models have comparable representations. The manuscript sometimes seems like two separate manuscripts: One set of results for sequential models only, and another for VAEs only. There is a section contrasting the two models, but I have questions about the comparison below. The authors have interesting results in each case separately, however.

This is a valid concern, which we have also frequently discussed internally while preparing the manuscript. Ultimately, we found that there is a need to consider these different models under the same lens, both because they are frequently used by practitioners under the same name (representation learning), but also because recent activity in the field is bringing them closer together, and the distinction is no longer clear cut. For instance, while some models are indeed fundamentally sequential (auto-regressive), transformers are often trained on a masked output loss, and can be viewed as Markov Random Fields (https://arxiv.org/abs/1902.04094), in principle making them interpretable using tools similar to what we discuss in the 2nd half of the paper. On the other hand, VAEs were originally proposed for training on aligned sequence of single protein families, but recent developments make it possible to train on unaligned sequences, opening up for possibilities to learn representations for multiple families (https://www.biorxiv.org/content/10.1101/2020.07.31.231381v1). We therefore opted for combining them into a single manuscript, such that we can highlight the similaries and differences. However, we agree with the overall concern, and in the new version of the manuscript, we have tried to further reduce the gap between the two parts of the manuscript.

In Figure 2, which is concerned with the interpretation aspects of representations, we now include the Bottleneck Resnet, which was introduced in the context of transfer learning, demonstrating that this choice has an impact on both perpectives. We've also restructured the initial sections on transfer learning so that there is a more natural progression. We now also explicitly discuss the dimensionality reduction step, and include a new figure in supporting information demonstrating the effect of this choice on the representation (contrasting t-SNE with the simple PCA method). Finally, as the reviewer suggested, we have updated Figure 1, so that it reflects both scenarios, thus integrating the two story lines more closely. We address the concerns regarding the comparisons between the models in detail below.

> *Specific questions:*
> *1. Contrasting sequential models with VAE*
> *The authors treat the sequential models and the VAE as comparable and generalize their discussion to make points about both simultaneously. But the two kinds of analysis may be more different than the authors allow.*
>
> *Tables 1 and 2, figs S1 S2 seem to apply only to the sequential models, while figures 3, 5, 6, 7 apply only to the VAE. The implications at lines 183, 217, 256 seem to only apply to sequential models (the VAE has no "pre-training"),*

Although we don't show examples of it in our manuscript, we would argue that there are many examples where you would describe the training of a VAE as a "pre-training" step. For instance, in other projects we use VAEs as the basis for optimizing functional traits of enzymes. In this setting, we often have hundreds to thousands of sequences available in the online sequence databases, but only tens to hundreds of experimental observations of the functional trait. We would then "pretrain" a VAE on the large number sequences, and then afterwards use the representation from the VAE as input to a regression algorithm trained on the small set of observations of the functional trait of interest. In the new version of the manuscript, we have added a paragraph discussing the difference between this global vs local scope of the representations (in "Representations for transfer learning").
Specifically to the figures you mention, we do believe that they carry relevance to both perpectives. E.g. the aggregation strategies discussed early in the paper have relevance to the interpretability, and e.g. Fig 6 shows how preprocessing of data can tune the representation towards particular parts of the input space, which is also relevant for sequential models in the transfer-learning setting (our results suggest that sequence reweighting should perhaps be considered as standard practice when training language models as well).

> *while the implications in lines 368, 469, 499 seem to apply only to the VAE-like models (the sequential models do not have a low-dim embedding, without t-SNE, see below).*

The geometric analysis we do is in principle not restricted to VAE-like models, but we agree that there is indeed an important distinction between models that learn a global representation as part of their reconstruction goal, and models that only learn local representations, which are then combined post-hoc to obtain global representations. In the new version of our manuscript, we now clearly make this distinction, and discuss the impact

of the post-hoc dimensionality reduction step, by comparing t-SNE with a simple PCA projection for the sequential models (Fig S3). We also train VAEs with higher dimensionality, and investigate the role that a dimensionality reduction step plays there (Fig S4).

> *As the authors discuss (lines 273-300), sequential models are typically applied to variable-length sequences from multiple protein families, while the VAE models are applied to aligned (equal length) sequences from a single protein family. In other words they typically apply to different kinds of data, though as the authors test the sequential model can also be applied to aligned sequences.*
>
> *But more importantly, the "latent-space" internal to the VAE seems quite different from the "local representation" of the sequential models. For the sequential models, the local representation is the "next/missing character" internal states, so that there are L (sequence length) separate local representations for each sequence. However, for the VAE model, each sequence is projected into a single 2-dimensional latent space. In other words, the VAE reduces the dimensionality of an input sequence (from L to 2) and embeds the sequence in the low-dim metric space, while the sequential models seem to increase the dimensionality of the representation (from L to L*4 for 4 local dimensions used in the manuscript, before aggregation) and the resulting set-of-local-representations do not explicitly have an interpretation as a metric space.*
>
> *The discussion contrasting the two models in lines 273-300 is relevant to the field and interesting. The authors conclude that "model architecture [has] a fundamental impact on the resulting representations", which I agree with, but should one then go further and say that the model architectures are different enough their internal representations are not directly comparable, and that only the VAE "embeds" the input into a lower dim space?*

We agree that this is an important distinction. We note that in the first part of the paper, about aggregation of sequences into a single representation, the "Bottleneck" and "Concat" strategies do actually both embed the sequences into a global representation, from which we can also decode. This representation is indeed much too high to be directly plotted in a visualization, but one could still interpret this as a metric space, and in principle calculate geodesics (although the high dimensionality might lead to technical issues).
As you mention, there are two issues at stake: the aggregation from L to a constant sized representation, and the reduction of that dimension. In the new version of the manuscript, we now investigate both these issues, placing greater emphasis on the dimensionality reduction step than we did originally. We now show that the t-SNE and PCA reveal similar patterns when reducing the dimension of the high dimensional representations from the sequential models (Fig S3) - and demonstrate that a VAE with a 30 dimensional latent space reproduces a (rough) star-like shape when reduced with PCA, but fails to do so with t-SNE.

> Some detailed comments on this distinction:
>
> i. In figure 2, the authors contrast the embedding of the sequential models (panels A-F) with that of the VAE (panel G). But I am not sure A-F are really lower-dim embeddings produced by the sequential models: The dimension reduction to 2 dims

was carried out after-the-fact using t-SNE. Is the lack of phylogenetic structure caused by the local-representation mapping, or by the dim reduction of t-SNE? The fact that the VAE latent space looks so different from the t-SNE result perhaps suggests that the latent space of the VAE is a different object conceptually than the local-representation-set of the sequential models. The same applies to Fig 4, left: I think this is based on the distances in the t-SNE 2d plot, so does this result arise because of t-SNE's representation or because of the sequential model representation?

Please see the discussion on the previous point. Our new results (Fig S3-S4) demonstrate that a simple PCA dimensionality reduction maintains a star shape like pattern when applied to a high dimensional VAE, while not doing so when applied to the output of the sequential models, suggesting that there is an actual difference in the learned representation. Regarding figure 4, we calculate the distance directly on the high dimensional representation, and thus do not rely on the t-SNE mapping for this plot. We acknowledge that this was not clear in the original paper and have now added a sentence to the caption of Fig 4 to clarify this.

ii. While figure 1 is meant to provide an overview of the methods, it shows only a transformer model with L local representations, and not a VAE. If this diagram were of a VAE, it would have only a single "local representation" which could be used to reconstruct the input. The "task model" and "global-to-local strategies" sections of figure 1 don't seem applicable to the VAE. This was confusing as I initially thought this was meant to show a VAE.

Thanks for pointing out this potential source for confusion. We have now modified Figure 1 to include both types of representations.

iii. The authors note in footnote 1 that "representation" and "embedding" are used interchangeably in the literature, but should they be? Embedding might be more appropriate for latent spaces with a metric.

We agree that it would make sense to distinguish between these two concepts, but fear that this battle might be lost since the terms are used too frequently in both settings in the literature.

iv. In table 1, it was not clearly stated exactly which model is being used. It is a sequential model since the authors imply L local representations in the text, but is it a transformer, LSTM, ResNet? Could the analysis of table 1 be done with the VAE?

This was indeed not clear. In the new version of the manuscript, we now state explicitly which model (and which architecture) was used for each experiment.

2. Comments on the Sequential model analysis

The analysis here (tables 1 and 2) is useful. The results that overtting can be prevented by fixing the model, and that adding an extra learning layer in task output

to improves predictions, might not be that surprising though, but perhaps that is because the authors explained the reason for the results so clearly and convincingly.

The methods used to make table 1 need to be explained, there are no methods or code provided for tables 1 or 2. The analysis was done building on the TAPE code, but additional detail could be given on the additions (eg, what is the autoencoder architecture for bottleneck?).

The lack of a clear description of the model architectures used in table 1 was indeed a mistake (and also commented on by the other reviewers). In this new version, we clearly state the models used for each experiment in the supplementary material, and we have now also made all code available, including the modifications we made to the TAPE respository, and additional notebook examples.

3. Comments on the VAE analysis

Some of the conclusions here do not seem entirely novel, but are new in the sense they are applied specifically in the context of protein sequnces and given context. Eg, fig 7 contrasting the paths in latent space is quite similar to (Ref 40: Arvanitidis 2018, fig 3), who discuss a similar figure saying "induced distances will be large in regions of the latent space where the generator is highly uncertain, these paths paths and distances. will then tend to follow the data in the latent space" (compare to line 607). Similarly, ref 40 says "In general, latent space distances [...] are sensitive to specifics of the underlying neural nets. It is therefore more robust to consider infinitesimal distances along the data manifold in the input space. [i.e, geodesics]" (compare to line 469 on robustness).

Indeed, there are connections to this earlier work by one of the co-authors of this current manuscript. However, the original publication looked only on toy-data sets, and on continuous data rather than the categorical data (amino acid output) that we consider here - which required us to consider entropy rather than variance as the underlying measure of uncertainty, and look at a different metric in output space. We therefore consider these results as novel.

Some other comments on the VAE:

Are figures 3 and 5 made using sequences weighted to account for density in sequence space using a sequence similarity cutoff? If such weighting is performed, it might erase much of the phylgenetic structure, which could lead to quite a different picture and to different geodesics. It would be interesting to test this using the reweighted data used in fig 6, in comparison.

Figure 3 and 5 were made without sequence reweighing, because the reweighting step was introduced later in the text. It is indeed interesting to investigate whether this has an impact on the correlation with phylogenetic distances. We have now redone the experiments on reweighted data (Fig S6). The star shape remains, but the branches that dominated the original representation become shorter, while branches that were underrepresented in the

original become more pronounced. The correlation is indeed slightly reduced, although there is still a benefit to using geodesics over Euclidian distances.

> *The results in fig 7 showing that the "unsupported" regions latent space (as in points 3,4,5 in the euclidean path in fig 7) yields high-entropy decodings has long raised questions to me about how VAEs behave. The VAE prior (a gaussian) suggests, perhaps, that these regions should correspond to valid sequences, and they should be sampled during the training/decoding process. Similarly, during the training process, why does the VAE not learn to map the data to a smooth gausian (the prior) in latent space, as should happen if the encoder properly maps to the prior? I am not sure if it is fair to ask the authors to explain these questions since they are deep questions about VAEs, but if the authors have any insights they would be good additions to the manuscript.*

These are indeed interesting questions. One could argue that the tree-like latent space that we observe when training a VAE on an alignment indicates that the model is not well specified. Using a different prior (e.g. a mixture of Gaussians or a hyperbolic prior) could potentially resolve this issue - at least to the extent that one could then sample meaningfully from the prior, and expect to decode to meaningful protein sequences. It would, however, not solve the issue that the latent space has a non-trivial geometry, and we thus considered the problems with prior specification as somewhat orthogonal to the qualities of the representation. However, it is certaintly a relevant problem. Your comment sparked a long debate about the topic and a discussion about whether to include it in the paper, but we ultimately decided against it, since it becomes a quite technical discussion specifically on VAEs, which we felt did not fit well with the scope of the paper.

> *Is it possible that there is some circularity in the results, eg of figure 3? The geodesics are constructed based on distances in the one-hot space, so should it be surprising that the geodesics match the phylogenetic distances well, since the phylogneetic distances are also based on distances in the one-hot space? Likewise this would explain why the geodesic distances are resilient to reparametrizations of the latent space.*

It is true that the geodesics are derived from a metric in output space, which in our case is the expected distance between one-hot vectors. One could therefore indeed argue that the correspondence to phylogenetic distances is not surprising. We would also not expect that the geodesics are necessarily *better* than if we just measured distances in output space. In the new version of the paper, we have added a column to Fig 4 showing the correlation results obtained using the expected Hamming distance between decoded points, which indeed has a slightly higher correlation with the phylogenetic distances. However, the most important aspect of the geodesics are that they take into account the uncertainty of the decoder, and thereby allows us to *interpolate* in latent space avoiding empty regions, which is what makes e.g. Fig 7 possible.

Reviewers' Comments:

Reviewer #1:
Remarks to the Author:
The authors successfully addressed all my comments.

I believe that the quality of their manuscript is now significantly improved by additional methods introduced into their comparison study about the pooling strategies. The authors also clarified some critical parts of their "bottleneck AutoEncoder" methodology and also significantly expanded the section on the geometry of the latent by adding mathematical notations that are now sufficiently clear, consistent and easy to follow.

Given the large number of available unlabelled sequences, and the importance of unsupervised and self-supervised model and their recent popularity in protein sciences, I believe that this manuscript presents many important challenges in sequence representation learning and provides useful guidance on how to use and interpret these representations that could be very useful in many future studies.

Reviewer #2:
Remarks to the Author:
The authors have made significant improvements to the manuscript, including improving descriptions of methodology and performing new experiments to address some of my previous concerns.

Overall, I think this is a good manuscript that will have impact on the community - I have personally taken some of the suggestions since first reviewing the paper and found that they do improve performance on unrelated downstream tasks.

The section I still have some trouble with is "Representation manifolds are shaped..." I think this section considers a few too many axes of variation simultaneously: We have model architecture, number of parameters, training data, and dimensionality reduction. For example, consider the Resnet Bottleneck architecture trained on beta-lac, compared with the VAE. These are very similar models. I believe the major differences are that the Resnet bottleneck has many more parameters, is trained on unaligned sequences, does not use the variational prior, and uses t-SNE for visualization. Because there are several changes, it's hard to determine the cause / impact of each change. The section still provides useful information and context, but it's a little too messy for me to draw very clear conclusions. That being said, the authors have showcased the most common approaches here, which may be good enough.

Minor Comments:
- In Table 1, it would be nice to describe the baseline in the caption.
- page 3, "echos" -> "echoes"
- supplementary page 2, "dilution" -> "dilation"

Reviewer #3:
Remarks to the Author:
The paper generally looks great and I have no substantive changes that I think are needed before publication.

However, there are several small things I want to bring to the authors attention. I believe the other reviewers are better qualified to address the neural network aspects of this paper, so I am focusing my attention on how well this paper meets its goal of being an introduction to NN for a wider biological

audience.

The biggest, most glaring shortcoming of the paper is how long it takes before the authors make clear what is meant by the word "representation." Representation of biological sequences is something that biologists have long considered (should a protein be represented by its protein sequence or its DNA sequence? should you describe its post-translational modifications? should it be in ASCII or binary? should you compress the sequence? should you store it on disk or tape or just resequence it when it's needed?) -- and so this absolutely ridiculous choice of words to make jargon (imagine the confusion arising from making the word "the" into jargon) needs to be addressed in the first sentence. It is not made clear until the second paragraph of the results section:

"The key idea is to leverage the many available unlabelled protein sequences to learn (pre-train) a general protein representation through an embedding model and then train a problem specific task model on top using the limited labelled training data"

A biologist will not make it to this paragraph before giving up.

The poor decision to turn "representation" into jargon is not something you should be faulted for, but it has a negative impact on this paper.

The introduction should be rewritten.

Besides this flaw, there are a lot of other poor word choices in the introduction:

"results have lingered" -- why would results have paused on a journey from one place to another? how does a result even move?
"an uprise in biology" -- is there a coup on the way? you mean a "rise."

paragraph 2: general properties of language that support *subsequent* specific

results paragraph 1: "patterns underlying data"
perhaps you mean "the data's underlying patterns"?

"we place our initial focus there and return later to data interpretation" -- you cannot return to something you have not visited the first time. How about "and turn later to data interpretation"?

I find "global scope" to be an odd phrase to use in NN.

In the subsection "fine-tuning can be detrimental to performance: "are conceptually considered separately" should become "are conceptually separate"

Page 6,
Start a new paragraph between "on a single protein family [4][35]." and "When training"

"the ability to perform such operations on proteins would have substantial impact on protein engineering and design" -- you should give an example here.

"However the lack of such results in the literature suggests that some aspects of leraned representations remain poorly understood"
OR it hasn't been tried. You can't publish "here's a protein sequence that came out of this neural network," without actually making that protein. Making proteins is an expensive, labor-intensive, and relatively specialized task and while there are plenty of people doing it, there are very few who also are versed in recent neural network results. The closest thing I've seen is by Anishchenko et al.: https://doi.org/10.1101/2020.07.22.211482 and it lacks the "we went and synthesized the protein"

data that they are likely in the process of generating.

Page 7: "for proteins this amount*s* to measuring latent"

Page 9: "biologially distances that are more meaningful" should be "biological distances"

Figure 7c: Label which column is the geodesic and which the straight-line euclidean. The text alludes to which is which, but ideally the reader should not have to read the text to pick up on the meaning of a figure.

Reviewer #4:
Remarks to the Author:
The authors have addressed many issues, and I think the manuscript is interesting and suitable for publication.

In the previous review my concern was that while the authors were usefully attempting to create a unified framework of conclusions about a class of machine learning models, the manuscript felt like two sets of results: One set for transformer-like models, another for VAEs, which only applied to one model and not the other because of fundamental differences in the two types of models.

The authors acknowledged differences between the two models but argue that the distinction is becoming less clear cut in literature, and have updated the manuscript to justify that the two types of models should be considered together. Reading the manuscript again, I think the theme of the importance of representation, encompassing both types models, is justified, and the manuscript provides an interesting unifying perspective even if some of the detailed conclusions are not universal to all models.

Some comments on the new content:

The authors responded to the questions about whether the post-hoc embedding method made the comparisons between the VAE vs transformer representations fair, by adding figures S3 and S4. These figures confirm their visualization results for transformers do not appear sensitive to the embedding method, at least, for two methods tSNE vs PCA. This further justifies the conclusions about differences in model's internal representations. Figure S4, for the VAE, to my eye suggests that the post-hoc embedding method has a significant effect on visualizations in figure 2, since the S4 plots for VAE with post-hoc emedding look fairly different from VAE plot in figure 2I without it. In other words, the differences in figure 2 might be ascribed more to the t-SNE post-hoc embedding method, than to the trained models' internal representation. However, the updated implication ("score of the data...") now explicitly mentions post-hoc dimensional reduction. While something about the issue of trained representation vs post-hoc embedding still seems unclear or unresolved to me, this is a detail in the wider context of the paper, and the updated implication makes sense and can stand as-is.

The authors tested the latent-space visualization for the VAE on phylogenetically reweighted data, as suggested. I asked this because I suspected that phylogenetic bias (non i.i.d. sequences) is a major cause of clustering in VAE latent space, so if this bias is removed the geodesics would look quite different. They find that this does reduce the clustering in latent space, though it does not eliminate it. As a comment (I'm not requesting further computation), I suspect this phylogenetic non-i.i.d. bias could be completely removed if the sequence identity cutoff is reduced from the 80% used here (from DeepSequence) to 40-50% (closer to the typical sequence identity between "random" evolutionarily i.i.d pairs of sequences in a pfam family), and that the geodesics would not correspond well to phylogeny. In any case, I appreciate the authors did this test, and the cutoff of 80% they appear to have used is well-enough justified as it is quite standard in literature.

Point-by-point response

We thank the reviewers for taking the time to review our revised manuscript. Below is a point-by-point response to each of the issues raised.

Reviewer #1 (Remarks to the Author)

> The authors successfully addressed all my comments.
>
> I believe that the quality of their manuscript is now significantly improved by additional methods introduced into their comparison study about the pooling strategies. The authors also clarified some critical parts of their "bottleneck AutoEncoder" methodology and also significantly expanded the section on the geometry of the latent by adding mathematical notations that are now sufficiently clear, consistent and easy to follow.
>
> Given the large number of available unlabelled sequences, and the importance of unsupervised and self-supervised model and their recent popularity in protein sciences, I believe that this manuscript presents many important challenges in sequence representation learning and provides useful guidance on how to use and interpret these representations that could be very useful in many future studies.

We thank the reviewer for the positive remarks.

Reviewer #2 (Remarks to the Author)

> The authors have made significant improvements to the manuscript, including improving descriptions of methodology and performing new experiments to address some of my previous concerns.
>
> Overall, I think this is a good manuscript that will have impact on the community - I have personally taken some of the suggestions since first reviewing the paper and found that they do improve performance on unrelated downstream tasks.

We thank the reviewer for the positive remarks.

> The section I still have some trouble with is "Representation manifolds are shaped..." I think this section considers a few too many axes of variation simultaneously: We have model architecture, number of parameters, training data, and dimensionality reduction. For example, consider the Resnet Bottleneck architecture trained on beta-lac, compared with the VAE. These are very similar models. I believe the major

> differences are that the Resnet bottleneck has many more parameters, is trained on unaligned sequences, does not use the variational prior, and uses t-SNE for visualization. Because there are several changes, it's hard to determine the cause / impact of each change. The section still provides useful information and context, but it's a little too messy for me to draw very clear conclusions. That being said, the authors have showcased the most common approaches here, which may be good enough.

This is a fair point. In some cases, the data preprocessing is tightly linked to the model architecture, which makes it difficult to fully separate the effect of the different contributions. For instance, the simple fully-connected architecture used in the VAE is only possible because the data has been aligned. In the specific case mentioned by the reviewer, we have experimented with various models on the spectrum that the reviewer describes. Unfortunately, several of such models cannot be trained due to the severity of the bottleneck. For instance, although it would have been ideal to limit our ResNet Bottleneck model to a two-dimensional representation space (avoiding the need for dimensionality reduction), we found it impossible to train such a model.
In this new version of the paper, we have expanded the section to include a discussion of the different contributions to representation quality, and the difficulty in disentangling the effects from them. We have also expanded the Supplementary Material with two new figures: 1) a new figure comparing the result obtained from a standard autoencoder compared to that of a variational autoencoder (which one one of the differences on the spectrum), and 2) a plot showing the representations obtained by a state-of-the-art language model (esm1b), which shows the affect of scaling up transformer models to many more parameters.

> Minor Comments:
> - In Table 1, it would be nice to describe the baseline in the caption.
> - page 3, "echos" -> "echoes"
> - supplementary page 2, "dilution" -> "dilation"

We have made the suggested corrections to the paper and thank the reviewer for pointing them out.

Reviewer #3 (Remarks to the Author)

> The paper generally looks great and I have no substantive changes that I think are needed before publication.

We thank the reviewer for the positive remarks.

> However, there are several small things I want to bring to the authors attention. I believe the other reviewers are better qualified to address the neural network aspects of this paper, so I am focusing my attention on how well this paper meets its goal of being an introduction to NN for a wider biological audience.

The biggest, most glaring shortcoming of the paper is how long it takes before the authors make clear what is meant by the word "representation." Representation of biological sequences is something that biologists have long considered (should a protein be represented by its protein sequence or its DNA sequence? should you describe its post-translational modifications? should it be in ASCII or binary? should you compress the sequence? should you store it on disk or tape or just resequence it when it's needed?) -- and so this absolutely ridiculous choice of words to make jargon (imagine the confusion arising from making the word "the" into jargon) needs to be addressed in the first sentence. It is not made clear until the second paragraph of the results section:

"The key idea is to leverage the many available unlabelled protein sequences to learn (pre-train) a general protein representation through an embedding model and then train a problem specific task model on top using the limited labelled training data"

A biologist will not make it to this paragraph before giving up.

The poor decision to turn "representation" into jargon is not something you should be faulted for, but it has a negative impact on this paper.

The introduction should be rewritten.

We thank the reviewer for taking the time to suggest ways to make our manuscript more accessible. We completely agree that "representation" is a terribly confusing part of the machine learning terminology. However, at this point "representation learning" is an established subfield of machine learning (perhaps due to this influential 2012 paper: https://arxiv.org/abs/1206.5538, cited almost 10000 times). It is therefore difficult to avoid these terms without confusing the machine learning part of our audience. However, we agree with the reviewer that this balancing act can be improved upon. In the revised manuscript we have restructured the introduction to already in the first sentences discuss the notion of “representation” and the estimation (learning) thereof.

Besides this flaw, there are a lot of other poor word choices in the introduction:

"results have lingered" -- why would results have paused on a journey from one place to another? how does a result even move?
"an uprise in biology" -- is there a coup on the way? you mean a "rise."

paragraph 2: general properties of language that support *subsequent* specific

results paragraph 1: "patterns underlying data"
perhaps you mean "the data's underlying patterns"?

"we place our initial focus there and return later to data interpretation" -- you cannot return to something you have not visited the first time. How about "and turn later to data interpretation"?

These changes have now been incorporated. We thank the reviewer for helping us sharpen up the language of our manuscript.

> I find "global scope" to be an odd phrase to use in NN.

We have now rephrased it so that we use "universal" for the representation models that are trained on all available protein data, and "family-specific" for those trained only on sequences from a single family.

> In the subsection "fine-tuning can be detrimental to performance: "are conceptually considered separately" should become "are conceptually separate"
>
> Page 6,
> Start a new paragraph between "on a single protein family [4][35]." and "When training"

We have incorporated these changes in the new version of the manuscript.

> "the ability to perform such operations on proteins would have substantial impact on protein engineering and design" -- you should give an example here.

We have now added an example to this sentence, suggesting that this would be useful for interpolating between biochemical properties or functional traits of a protein.

> "However the lack of such results in the literature suggests that some aspects of leraned representations remain poorly understood"
> OR it hasn't been tried. You can't publish "here's a protein sequence that came out of this neural network," without actually making that protein. Making proteins is an expensive, labor-intensive, and relatively specialized task and while there are plenty of people doing it, there are very few who also are versed in recent neural network results. The closest thing I've seen is by Anishchenko et al.: https://doi.org/10.1101/2020.07.22.211482 and it lacks the "we went and synthesized the protein" data that they are likely in the process of generating.

This is a valid point. In our discussions with colleagues across the field, and an active research collaboration with a company producing enzymes through ML-driven engineering based on predictions, our experience is that current tools do not have the capability to interpolate meaningfully between proteins, in particular when these are separated by more than a few mutations. This is what we attempted to convey with our original sentence - that it is an open question. However, we agree with your comment that we don't have a full overview over how much effort has gone into this. We have therefore decided to remove the sentence and now conclude the paragraph with a question: "What is required of our representations to support such interpolations?"

> Page 7: "for proteins this amount*s* to measuring latent"

Page 9: "biologially distances that are more meaningful" should be "biological distances"

Figure 7c: Label which column is the geodesic and which the straight-line euclidean. The text alludes to which is which, but ideally the reader should not have to read the text to pick up on the meaning of a figure.

The new version of the manuscript incorporates these changes.

Reviewer #4 (Remarks to the Author)

The authors have addressed many issues, and I think the manuscript is interesting and suitable for publication.

In the previous review my concern was that while the authors were usefully attempting to create a unified framework of conclusions about a class of machine learning models, the manuscript felt like two sets of results: One set for transformer-like models, another for VAEs, which only applied to one model and not the other because of fundamental differences in the two types of models.

The authors acknowledged differences between the two models but argue that the distinction is becoming less clear cut in literature, and have updated the manuscript to justify that the two types of models should be considered together. Reading the manuscript again, I think the theme of the importance of representation, encompassing both types models, is justified, and the manuscript provides an interesting unifying perspective even if some of the detailed conclusions are not universal to all models.

We thank the reviewer for the positive remarks.

Some comments on the new content:

The authors responded to the questions about whether the post-hoc embedding method made the comparisons between the VAE vs transformer representations fair, by adding figures S3 and S4. These figures confirm their visualization results for transformers do not appear sensitive to the embedding method, at least, for two methods tSNE vs PCA. This further justifies the conclusions about differences in model's internal representations. Figure S4, for the VAE, to my eye suggests that the post-hoc embedding method has a significant effect on visualizations in figure 2, since the S4 plots for VAE with post-hoc emedding look fairly different from VAE plot in figure 2I without it. In other words, the differences in figure 2 might be ascribed more to the t-SNE post-hoc embedding method, than to the trained models' internal representation. However, the updated implication ("score of the data...") now explicitly mentions post-hoc dimensional reduction. While something about the issue of

trained representation vs post-hoc embedding still seems unclear or unresolved to me, this is a detail in the wider context of the paper, and the updated implication makes sense and can stand as-is.

The authors tested the latent-space visualization for the VAE on phylogenetically reweighted data, as suggested. I asked this because I suspected that phylogenetic bias (non i.i.d. sequences) is a major cause of clustering in VAE latent space, so if this bias is removed the geodesics would look quite different. They find that this does reduce the clustering in latent space, though it does not eliminate it. As a comment (I'm not requesting further computation), I suspect this phylogenetic non-i.i.d. bias could be completely removed if the sequence identity cutoff is reduced from the 80% used here (from DeepSequence) to 40-50% (closer to the typical sequence identity between "random" evolutionarily i.i.d pairs of sequences in a pfam family), and that the geodesics would not correspond well to phylogeny. In any case, I appreciate the authors did this test, and the cutoff of 80% they appear to have used is well-enough justified as it is quite standard in literature.

We thank the reviewer for his comments regarding our last revision. Although he did not request further computation we were curious ourselves and have actually added just one more experiment to shed light on this last issue: we have now trained models at cutoff levels 0.2, 0.4, 0.6, 0.8, and show the impact of this cutoff on both the embedding and the correlation coefficient (Supplementary Figure 8).
